# Comparative genomic and phenotypic analyses of the virulence potential in Shiga toxin-producing *Escherichia coli* O121:H7 and O121:H10

**DOI:** 10.3389/fcimb.2022.1043726

**Published:** 2022-11-24

**Authors:** Michelle Qiu Carter, Nicole Laniohan, Antares Pham, Beatriz Quiñones

**Affiliations:** Produce Safety and Microbiology Research Unit, Western Regional Research Center, Agricultural Research Service, U.S. Department of Agriculture, Albany, CA, United States

**Keywords:** Shiga toxin-producing *Escherichia coli*, comparative genomics, Shiga toxins, O121, virulence, pathogenicity islands, cytotoxicity, Stx-prophages

## Abstract

Shiga toxin-producing *Escherichia coli* (STEC) O121 is among the top six non-O157 serogroups that are most frequently associated with severe disease in humans. While O121:H19 is predominant, other O121 serotypes have been frequently isolated from environmental samples, but their virulence repertoire is poorly characterized. Here, we sequenced the complete genomes of two animal isolates belonging to O121:H7 and O121:H10 and performed comparative genomic analysis with O121:H19 to assess their virulence potential. Both O121:H7 and O121:H10 strains carry a genome comparable in size with the O121:H19 genomes and belong to phylogroup B1. However, both strains appear to have evolved from a different lineage than the O121:H19 strains according to the core genes-based phylogeny and Multi Locus Sequence Typing. A systematic search of over 300 *E. coli* virulence genes listed in the Virulence Factor DataBase revealed a total of 73 and 71 in O121:H7 and O121:H10 strains, respectively, in comparison with an average of 135 in the O121:H19 strains. This variation in the virulence genes repertoire was mainly attributed to the reduction in the number of genes related to the Type III Secretion System in the O121:H7 and O121:H10 strains. Compared to the O121:H19 strains, the O121:H7 strain carries more adherence and toxin genes while the O121:H10 strain carries more genes related to the Type VI Secretion System. Although both O121:H7 and O121:H10 strains carry the large virulence plasmid pEHEC, they do not harbor all pEHEC virulence genes in O121:H19. Furthermore, unlike the O121:H19 strains, neither the O121:H7 nor O121:H10 strain carried the Locus of Enterocyte Effacement, OI-122, nor the tellurite resistance island. Although an incomplete Locus of Adhesion and Autoaggregation (LAA) was identified in the O121:H7 and O121:H10 strains, a limited number of virulence genes were present. Consistently, both O121:H7 and O121:H10 strains displayed significant reduced cytotoxicity than either the O157:H7 strain EDL933 or the O121:H19 strain RM8352. In fact, the O121:H7 strain RM8082 appeared to cause minimal cytotoxicity to Vero cells. Our study demonstrated distinct evolutionary lineages among the strains of serotypes O121:H19, O121:H10, and O121:H7 and suggested reduced virulence potentials in STEC strains of O121:H10 and O121:H7.

## 1 Introduction

Shiga toxin-producing *Escherichia coli* (STEC) consists of a group of genetically and phenotypically diverse bacterial strains differing greatly in pathogenicity. Such variation is attributed in part to the differences in their genetic makeup, especially the repertoire of virulence genes. The genomes of *E. coli* are extremely flexible, consisting of a core genome that is conserved among the *E. coli* strains, and an accessory genome containing strain, lineage, or pathovar-specific genes (Rasko et al., 2008; Touchon et al., 2009; Chaudhuri and Henderson, 2012; Robins-Browne et al., 2016). Enterohaemorrhagic *Escherichia coli* (EHEC) refer to a subset of STEC associated with severe human illnesses including bloody diarrhea and hemolytic uremic syndrome (HUS). The classical characteristics of EHEC include the production of Shiga toxin (Stx), formation of attaching-and-effacing (A/E) lesions on intestinal epithelial cells, and production of enterohemolysin (Nataro and Kaper, 1998). Genes encoding these virulence factors are all located on mobile genetic elements (MGEs), including prophages for Shiga toxins, the locus of enterocyte effacement (LEE) for the A/E lesion and type III secretion system (T3SS), and a large virulence plasmid (pEHEC) for enterohemolysin production. However, atypical EHEC strains have evolved, such as the STEC O104:H4 linked to the 2011 large outbreak of hemorrhagic diarrhea in Europe (Rasko et al., 2011). This O104:H4 outbreak strain lacks LEE but harbors the other virulence genes with a comparable function to the LEE-encoded intimin in the plasmid pAA, which confers a super aggregative capability and promotes the adherence of bacteria to the host epithelial cells (Bielaszewska et al., 2011a; Rasko et al., 2011).

In addition to LEE and pEHEC, there are several genomic islands (GIs) and pathogenicity islands (PAIs) in *E. coli* strains conferring advantages in all aspects of survivability in diverse ecological niches, including pathogenicity, nutrient uptake, metabolism, and stress resistance. The Acid Fitness Island (AFI), first reported in *E. coli* K-12 strain MG1655 (Mates et al., 2007), is a 14-kb GI providing the bacteria the ability to survive under extreme acidic conditions. The Locus of Heat Resistance (LHR), a 15-kb GI that was first discovered in a heat resistant *E. coli* food isolate (Mercer et al., 2015), confers resistance to heat, chlorine, and oxidizing agents (Mercer et al., 2017). Tellurite resistance in STEC is mediated by a gene cluster comprising *terZABCDEF* that is co-located with the gene encoding the Iha adhesin on a large GI known as tellurite resistance- and adherence-conferring island (TRI) (Tarr et al., 2000). Great genetic diversity was reported for TRI in STEC populations (Taylor et al., 2002). In strain EDL933, there are two identical copies of the *ter* gene clusters, located on the O-islands #43 (OI-43) and #48 (OI-48) (Perna et al., 2001). Both OI-43 and OI-48 display chromosomal instability and could undergo complete excision by site-specific recombination between the direct repeats (DRs) flanking OI-43 or OI-48, resulting in intrapopulation variation in adherence and tellurite resistance (Bielaszewska et al., 2011b). Other EDL933 O islands that have been reported to play a role in pathogenicity of STEC included OI-57 and OI-122. The OI-57 contains several virulence genes including *adfO* and *ckf*, which encodes an adhesin and a phage-associated killer protein, respectively. The OI-57 is associated with STEC strains belonging to highly pathogenic serotypes (Imamovic et al., 2010). In fact, several genes on OI-57 have been proposed as molecular markers for diagnosis of typical EHEC and newly emerged EHEC strains (Delannoy et al., 2013). The OI-122 encodes a few non-LEE effectors, and in some STEC strains OI-122 and LEE are cointegrated (Konczy et al., 2008). The OI-122 is a virulence marker because it is often associated with the STEC strains capable of causing epidemic diseases (Wickham et al., 2006).

More recently, a large GI known as the Locus of Adhesion and Autoaggregation (LAA) was revealed in a subset of LEE negative STEC strains, and some of these strains could cause hemorrhagic colitis or HUS (Montero et al., 2017). The complete LAA contains four modules encoding virulence factors that can mediate colonization of bacteria to human epithelial cells and contributes to intestinal colonization of STEC in a mouse model (Montero et al., 2019). A complete LAA or an incomplete LAA (with less than four modules) is present in diverse serotypes of STEC (Montero et al., 2017; Vélez et al., 2020). Similarly, the subtilase encoding (SE)-PAI is primarily found in LEE negative STEC strains. This PAI contains genes encoding the SubAB cytotoxin and the gene (*tia*) encoding an invasion determinant that is primarily present in enterotoxigenic *E. coli* (Michelacci et al., 2013). Once the SubAB cytotoxin is delivered into the host’s endoplasmic reticulum, it cleaves the chaperone BiP, resulting in transient inhibition of host protein synthesis and subsequently the induction of apoptotic signaling pathways (Chong et al., 2008; Wyrsch et al., 2020). The locus of proteolysis activity (LPA) was named based on the protease activity of one of the proteins that LPA encoded, EspI (Schmidt et al., 2001). This PAI is typically found in LEE negative STEC strains, utilizing the same chromosomal integration site, *selC*, as the LEE in strain EDL933. The high pathogenicity island (HPI) is involved in iron storage and uptake and was originally identified in the *Yersinia* species (Schubert et al., 1998). The HPI has been reported present in diverse STEC clinical isolates (Karch et al., 1999).

STEC O121 is among the top six non-O157 serogroups that are most frequently associated with severe disease in humans (Brooks et al., 2005; Gould et al., 2013; Luna-Gierke et al., 2014). Outbreaks of STEC O121 infection have been reported worldwide with a HUS rate ranging from 7.1% to 27.3% (McCarthy et al., 2001; Yatsuyanagi et al., 2002; Luna-Gierke et al., 2014; Crowe et al., 2017; Morton et al., 2017; Boyd et al., 2021). The serotype O121:H19 is one of the non-O157 serotypes most frequently associated with HUS (Kappeli et al., 2011), and the predominant serotype in the STEC O121 environmental isolates (Carter et al., 2019). While population structure and the evolution of virulence in O121:H19 has been studied extensively (Nishida et al., 2021), little is known about the genetic diversity and virulence potential of other O121 serotypes. In this study, we sequenced the genomes of two animal strains that belong to O121:H7 and O121:H10, which are of the serotypes that have been frequently isolated in environmental samples (Cooley et al., 2007). To our knowledge, the two genomes reported in this study are the first complete genomes of the serotypes O121:H7 and O121:H10. We further performed comparative genomic analysis with the serotype O121:H19 to evaluate the pathogenicity potential and health risk of the STEC O121:H7 and O121:H10 strains.

## 2 Material and methods

### 2.1 Bacterial strains and growth media

Bacterial strains and their sources are listed in **Table 1**. The two animal strains (RM8082 and RM10740) were isolated as described previously (Cooley et al., 2007) and grown routinely in Luria-Bertani (LB) broth.

**Table 1:** *E. coli* strains used in this study and relevant genomic information.

Strains	^a^Sources(Location, year)	^b^Serotypes	^c^Phylogroups/Genotypes	*stx* genes	Chromosome (bp)/GenBank Accession Number	^d^Plasmids (bp)/GenBank Accession #		References
						pEHEC	Others	
EDL933	Ground beef (USA, 1982)	O157:H7	E/ST11	*stx*_1a_+*stx*_2a_	5,528,445/ AE005174.2	92,077/ AF074613.1	N/A	(Burland et al., 1998; Perna et al., 2001)
MG1655	Stool (USA, 1922)	O-:H48:K-	A/ST10	N/A	4,641,652/ U00096.3	N/A	N/A	(Blattner et al., 1997)
16-9255	Clinical (Canada, 2016)	O121:H19	B1/ST655	*stx*_2a_	5,397,521/ CP022407	81,950/ CP022408	NA	(Robertson et al., 2018)
2015C-3107	Clinical (USA, 2014)	O121:H19	B1/ST655	*stx*_2a_	5,388,260/ CP027317	81,954/ CP027318	NA	(Patel et al., 2018)
RM8352	Sediment (USA, 2009)	O121:H19	B1/ST655	*stx*_2a_	5,391,064/ CP028110	83,211/ CP028111	NA	(Parker et al., 2018)
RM8082	Cattle (USA, 2009)	O121:H7	B1/ST5082	*stx*_1d_	5,153,748/ CP043825	146,074/ CP043829	37,652/CP043826 94,550/CP043827 97,743/CP043828	This study
RM10740	Feral pig (USA, 2009)	O121:H10	B1/ST641	*stx*_2e_	5,111,631/ CP043821	114,358/ CP043824	39,610/CP043822 100,386/CP043823	This study

a The source and isolation year for strain MG1655 is based on the information available for its parental strain K-12 as described previously (Bachmann, 1996).

b The serotype of strain MG1655 is based on the information available for its parental strain K-12 as described previously (Liu and Reeves, 1994).

c The Genotype refers to the Sequence Type (ST) based on the Achtman scheme.

d pEHEC refers to the plasmid carrying genes encoding the enterohemolysin (*hlyCABD*). N/A, not present.

### 2.2 Genome sequencing, annotation, and comparative genomic analysis

Bacterial DNA was extracted from the mid-exponential phase cultures grown in LB broth as described previously (Miller et al., 2005) with slight modification. Briefly, cells were lysed with SDS followed by sequential treatment with RNase A and proteinase K. The DNA was first precipitated in a sodium acetate/ethanol solution, and then purified by phenol/chloroform extraction, followed by the final ethanol precipitation. The purified DNA was re-suspended in Qiagen Buffer Elution Buffer (QIAGEN) for genome sequencing. Single Molecule Real-Time (SMRT) sequencing was performed on a PacBio RSII instrument (Pacific Biosciences) using the protocol “Procedure & Checklist Greater than 10 kb Template Preparation Using AMPure PB Beads” followed by performing template binding using P6v2 sequencing Polymerase and Magbeads (Pacific Biosciences). The SMRTbell sequencing libraries were prepared using eight µg of sheared DNA and SMRTbell Template Prep Kit 3.0. The SMRT cells were run with 0.1 nM on-plate concentration, P6/C4 sequencing chemistry, MB1percv1 collection protocol, and 360-min data collection mode. A FASTQ file was generated using SMRT Analysis (v2.3.0) software, and the sequence reads were assembled into contigs with RS_HGAP_Assembly.3 software. The complete genome sequences were submitted to GenBank for annotation using Prokaryotic Genome Annotation Pipelines. The GenBank accession numbers are listed in **Table 1**. The phylogroups were determined using the Clermont method (Clermont et al., 2015). The Multi-Locus Sequence Typing (MLST) was conducted using MLST 2.0 service at the Center for Genomic Epidemiology with the *Escherichia coli* #1 configuration (Achtman 7 gene scheme, *adk*, *fumC*, *gyrB*, *icd*, *mdh*, *purA*, and *recA*) (Wirth et al., 2006). The genomic subsets, including the core genome, pangenome, and accessory genes, were calculated using Edgar 3.0 using the default setting (Blom et al., 2016; Dieckmann et al., 2021).

### 2.3 Identification of virulence genes

The putative virulence genes were identified using VFanalyzer at The Virulence Factor DataBase (VFDB) using pre-annotated genomes in GenBank format. Each pre-annotated genome was uploaded to VFanalyzer to scan for all *Escherichia* Virulence Factors (VFs) listed in VFDB as of April 2022, which includes genes identified in 37 *E. coli* strains belonging to adherent invasive *E. coli* (AIEC), avian pathogenic *E. coli* (APEC), enteroaggregative *E. coli* (EAEC), Shiga toxin-producing enteroaggregative *E. coli* (StxEAEC), EHEC, enteropathogenic *E. coli* (EPEC), enterotoxigenic *E. coli* (ETEC), neonatal meningitis-associated *E. coli* (NMEC), or uropathogenic *E. coli* (UPEC). The identified virulence genes were further verified by a BLASTn search of a database containing all STEC strains examined in this study in Geneious Prime® with a threshold of 80% for gene coverage and 70% for sequence identity. Genes carrying a loss-of-function mutation in the coding region were marked as being absent.

### 2.4 Detection of GIs and PAIs

GIs were first revealed using IslandViewer 4 (Bertelli et al., 2017) using the *E. coli* K-12 strain MG1655 genome as a reference. A GI was called when a prediction was made by at least one of the three methods (IslandPath-DIMOB, SIGI-HMM, and IslandPick). The GIs and PAIs known to contribute to stress resistance and/or pathogenicity in enteric pathogens (**Supplementary Table 1**) were further examined by performing BLAST searches against a custom database containing all genomes described in this study. When a complete GI or PAI was not detected, each CDS encoded by the query GI or PAI was used to search the genome of the testing strain by BLASTP to reveal if any homologs were present in the genome of the testing strain. Presence of key virulence genes on each PAI were revealed by BLASTn of each virulence gene followed by mapping their chromosomal locations in Geneious Prime®. Strain EDL933 harbors two TRIs, OI-43 and OI-48, which exhibit over 99% identity. The OI-48 was used as the query to search for homologs of TRI in other strains.

### 2.5 Analysis of Stx-prophages and virulence plasmid pEHECs

The complete genome sequence of each strain was submitted to PHASTER (Arndt et al., 2016) for identification of prophage and prophage-like elements. The putative integration sites were initially identified by PHASTER and confirmed in Geneious Prime® using “Find Repeats” in the defined chromosomal regions. Comparative analysis of pEHEC were carried out by BLASTP of all CDS encoded by each pEHEC against a custom database containing all genomes described in this study. A CDS was called positive when the hits have 80% for coverage and 25% for sequence identity at protein level. The genomic location of each CDS was mapped in Geneious Prime®.

### 2.6 Cytotoxicity assay

The Stx activity of the STEC O121 strains was measured using a mammalian Vero cell line, Vero-d2EGFP, that harbored a destabilized variant (t_1/2_ = 2 hr) of the enhanced green fluorescent protein (EGFP) as described previously (Quiñones et al., 2009; Quiñones and Swimley, 2011). Strain EDL933 and K-12 strain MG1655 were used as a positive and negative controls for the assay, respectively. Cell-free culture supernatants for all tested STEC strains were prepared as previously described (Quiñones et al., 2009; Quiñones and Swimley, 2011; Silva et al., 2019). One-day prior to intoxication, Vero-d2EGFP cells were seeded at 9,500 cells per well in Greiner Bio-One CELLSTAR^®^ black 96-well microplates with µClear^®^ bottoms (VWR International) and were grown at 5% CO_2_ and 37°C under humidified conditions in Ham’s F-12 Nutrient medium (Life Technologies Corp.), supplemented with 10% fetal bovine serum (American Type Culture Collection) and 1% penicillin-streptomycin (Life Technologies). The Vero-d2EGFP cells were then exposed to Ham´s F-12 medium containing tenfold dilutions of cell-free culture supernatants from each bacterial strain and were incubated at 37°C for 20 hr in a 5% CO_2_ humidified incubator. Following intoxication, the Vero-d2EGFP cells were briefly rinsed three times with 1X phosphate-buffered saline, and the EGFP fluorescence from the Vero-d2EGFP cells was measured using a BioTek Synergy HT Multi-Detection Microplate Reader (Agilent Technologies) with the 485/20 nm excitation filter and the 528/20 nm emission filter. All experiments were performed in triplicate and each experimental condition consisted of six replicates. The results were expressed as percentages of the GFP fluorescence values for culture supernatant-treated Vero-d2EGFP cells when compared to the fluorescence values from control Vero-d2EGFP cells incubated without bacterial supernatants. The significant differences in the cytotoxicity levels among the STEC strains were determined using the two-tailed test function (*P*-value < 0.05) in R version 4.2 (R Core Team 2022).

## 3 Results

### 3.1 Genomic characteristics of STEC O121:H7 strain RM8082 and O121:H10 strain RM10740

The STEC O121 genomes currently available in public databases are predominantly represented by strains belonging to the serotype O121:H19. To gain insights into the genetic diversity and virulence potential of STEC serogroup O121, the complete genomes of two animal isolates, recovered from a major agricultural region in the United States and belonging to serotype O121:H7 and O121:H10, respectively, were sequenced and annotated (**Table 1**). The genome of O121:H7 strain RM8082 was composed of a 5,153,748-bp chromosome and four plasmids, ranging in size (bp) from 37,652 to 146,074. The genome of O121:H10 strain RM10740 was composed of a 5,111,631-bp chromosome and three plasmids, ranging in size (bp) from 39,610 to 114,358. Comparative genomic analyses were performed with three representative O121:H19 genomes retrieved from GenBank, including strain 16-9255, linked to the 2016 Canadian flour-associated outbreak, strain 2015C-3107, a clinical isolate, and strain RM8352 a sediment isolate recovered from the same geographical region as strains RM8082 and RM10740 (**Table 1**).

**Figure 1:**
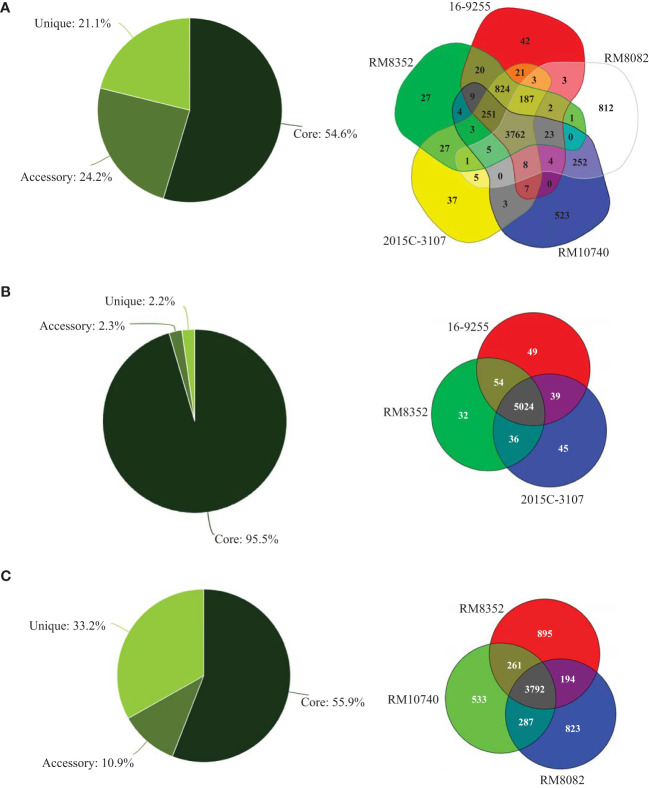
Comparative genomic analyses of STEC O121. Genomic subset distribution and Venn diagrams of **(A)** five STEC O121 strains, **(B)** three STEC O121:H19 strains, and **(C)** three non-clinical STEC O121 strains. The total genes are 6890 **(A)**, 5267 **(B)**, and 6785 **(C)** when the genome of strain RM8352 was used as a reference in all analyses. The numbers in the colored regions are the number of genes either unique to or shared by the corresponding strains. The analyses were performed in Edgar 3.0 using default parameters as detailed in the Material and Methods section. Core refers to the genes shared by all strains; accessory refers to the genes shared by at least two but not all strains; unique refers to the genes only present in one strain.

The chromosomes of both RM8082 and RM10740 were smaller in size than any of the O121:H19 chromosomes, however, the virulence plasmid pEHEC in both strains were larger in size than any of the O121:H19 plasmid pEHEC. Like the O121:H19 strains, RM8082 and RM10740 belonged to phylogroup B1, however, both differed from the O121:H19 strains and from each other based on the MLST typing (**Table 1** and **Figure 1**). Among the seven housekeeping loci examined, *recA* was the only common locus between strain RM10740 and the three O121:H19 strains, while no common loci were detected between strain RM8082 and the three O121:H19 strains. The five STEC O121 strains carried a total of 6,906 genes, of which, 54.6% were conserved, 24.2% were shared by at least two strains, and 21.1% were only detected in one strain (**Figure 1A**). This notable genetic diversity appeared to be attributed mainly to the O121:H7 and O121:H10 strains since the three O121:H19 strains shared a large core gene set (95.5%) (**Figure 1B**). In fact, the three non-clinical O121 strains (RM8082, RM8352, and RM10740) that were isolated from the same geographical region had a total of 6,785 genes, of which, 55.9% were conserved in all three strains, 10.9% shared by two strains, and 33.2% was only detected in one strain (**Figure 1C**). Consistent with the genotype or serotype-based strain relatedness, the core genome-based phylogeny placed all three O121:H19 strains in the same cluster, whereas RM8082 and RM10740 appeared to have evolved from separate lineages (**Supplementary Figure 1**).

### 3.2 Stx-prophages

**Figure 2:**
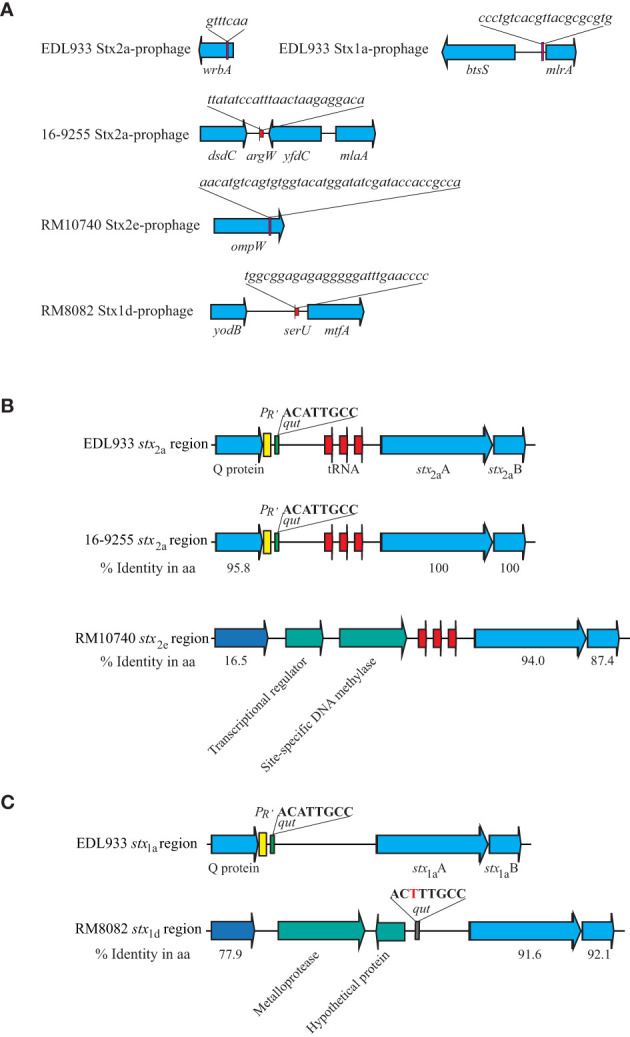
Sequence analyses of Stx-prophages in strains of STEC O121. **(A)** Chromosomal locations of Stx-prophages. The Stx-prophages and the putative integration sites were identified using PHASTER. The chromosomal locations of Stx-prophages are shown in schematic maps. Genes are not drawn to scale. Red arrows indicate tRNA genes, and the pink blocks represent the integration sites. **(B)** Sequence analyses of the *stx*_2_ regions. The % Identity is the percent identity in protein sequences in the pairwise comparison using the corresponding EDL933 protein as a reference. The sequences in the three STEC O121:H19 strains are identical. Red arrows indicate tRNA genes; yellow blocks refer to the late promoter *P*_R’_; and the green blocks refer to the *qut* site. **(C)** Sequence analyses of the *stx*_1_ regions. % Identity is the percent identity in protein sequences in the pairwise comparison using the EDL933 protein as a reference. The yellow block refers to the late promoter *P*_R’_; the green block refers to the *qut* site; and the grey block refers to an altered *qut* site.

Strain RM10740 (O121:H10) carried a Stx2e-prophage inserted within the gene *ompW* (**Figure 2A**). This integration event resulted in duplicating the 126-bp C terminal fragment of *ompW* coding sequence that bordered the Stx2e-prophage. Strain RM8082 (O121:H7) carried a Stx1d-prophage inserted in the tRNA gene *serU*. All three O121:H19 strains carried a Stx2a-prophage inserted in the tRNA gene *argW*. Both the Stx2e-prophage (~ 48 kb) and the Stx1d-prophage (~ 47 kb) were smaller in size than any of the Stx2a-prophages (~ 66-70 kb) in the O121:H19 strains (**Table 2**). Phylogenetic analysis of the O121 Stx-prophages with the Stx-prophages in O157:H7 strain EDL933 revealed two clusters. One cluster contained the EDL933 Stx1a-prophage and all Stx2a-prophages, and the other cluster contained the Stx2e-prophage and the Stx1d-prophage (**Supplementary Figure 2**). BLAST search of the Stx2e-prophage genome in the NCBI non-redundant (nr) database revealed homologs in several *E. coli* strains isolated from diverse sources, including pigs, sheep, freshwater samples, and clinical samples collected worldwide. For example, the first 31-kb segment of the Stx2e-prophage genome in strain RM10740 exhibited over 99% sequence identity with the Stx2e-prophage in *E. coli* strain RHB41-C11 that was isolated from a pig farm in the United Kingdom (GenBank accession number: CP056985.1, chromosome location: 2,867,972-2,899,718). Although homologs of the Stx1d-prophage late region were identified in multiple *E. coli* strains isolated worldwide, the region containing the *stx*_1d_ coding sequence and its regulatory elements were only identified in a few strains, including C311, a cattle isolate, that originated from Hong Kong in 2018 (GenBank accession number, CP062204.1), 2014C-3075, a clinical isolate that originated from the U.S. in 2013 (GenBank accession number, CP027447.1), and FHI40, a clinical isolate that originated from Norway in 2009 (GenBank accession number, LM996246.1).

**Table 2:** Genomic characteristics and chromosomal locations of Stx-prophages in STEC O121.

Strains (Serotypes)	^a^Stx-prophages				
	*stx* subtype	^b^Chromosomal locations	Size (bp)	%GC	CDS
16-9255 (O121:H19)	*stx*_2a_	*argW* -(5,357,665-5,397521; 1-30,182*)- yfdC*	70,039	48.7	95
2015C-3107 (O121:H19)	*stx*_2a_	*argW* -(1,479,827-1,411,104*)- yfdC*	68,724	49.6	89
RM8352 (O121:H19)	*stx*_2a_	*argW* -(3,914,192-3,980,289*)- yfdC*	66,098	49.5	90
RM8082 (O121:H7)	*stx*_1d_	*serU* -(2,233,920-2,186,865*)- yodB*	47,056	50.4	66
RM10740 (O121:H10)	*stx*_2e_	*ompW*-(1,446,065-1,494,043)- ompW*	47,979	50.5	69

a Prophages were determined using PHASTER (Arndt et al., 2016).

b The genome of the Stx2a-prophage in strain 16-9255 was corrected manually based on the sequences of attL and attR annotated. The region shown here was annotated containing two prophages by PHASTER (prophage 1 and Prophage 22). *Truncated genes.

The DNA segments containing the regulatory elements of the *stx*_2a_ in the three O121:H19 strains were nearly identical and highly similar to the *stx*_2a_ and *stx*_1a_ regulatory regions in strain EDL933 (**Figures 2B, C**). Specifically, all three O121:H19 strains harbored an antitermination protein Q of 144 amino acids that exhibits >95% sequence identity with the Q proteins in strain EDL933. Furthermore, both late promoter *P*_R’_ and the *qut* site are conserved in all three O121:H19 strains. In contrast, the O121:H10 strain RM10740 had an antitermination protein of 184 amino acids that exhibits 16.5% sequence identity with the Q protein encoded by EDL933 Stx2a-prophage. Neither a putative *P*_R’_ nor a *qut* site was identified in the upstream of the *stx*_2e_ coding sequence. Rather, two CDSs, encoding a TrmB family transcriptional regulator and a site-specific DNA methylase, respectively, were identified between the coding sequences of the antitermination protein gene and the *stx*_2e_. The Stx1d-prophage in strain RM8082 contained an antitermination protein of 123-amino acids, exhibiting 77.9% identity with the antitermination protein Q encoded by the EDL933 Stx1a-prophage. Although no putative *P*_R’_ was identified in the genome of the Stx1d-prophage, a putative *qut* site was identified upstream of the *stx*_1d_ coding sequence (**Figure 2C**).

### 3.3 Conservation of *E. coli* virulence genes in STEC O121 strains

**Figure 3:**
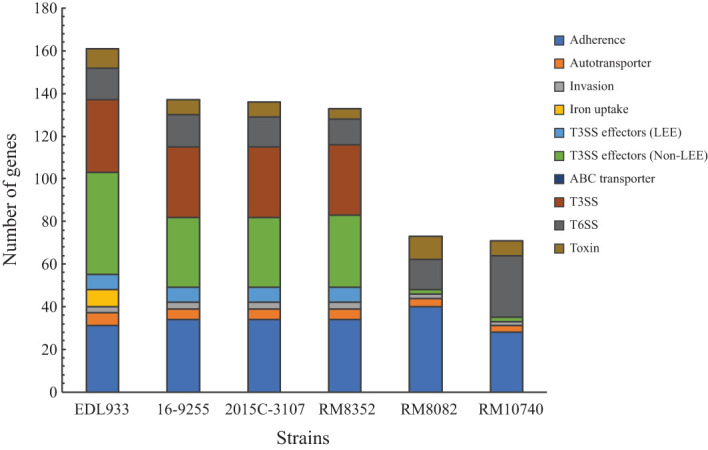
Detection of the *E. coli* virulence genes in STEC O121. The functional categories of the detected virulence genes are color-coded. The list of genes, their products and associated *E. coli* pathotypes are presented in **Supplementary Table 2**. Presence of each virulence factor in each STEC strain was verified by BLASTn search of a database containing all STEC strains in Geneious Prime^®^ with a threshold of 80% for coverage and 70% for sequence identity.

As shown in previous studies, a total of 333 *E. coli* virulence genes, listed in the VFDB were found to belong to 10 major functional categories and contribute to *E. coli* pathogenicity in diverse pathotypes (Carter et al., 2022). Of the 333 genes examined in the present study, a total of 137, 136, and 133 virulence genes were detected in the O121:H19 strains 16-9255, 2015C-3107, and RM8352, respectively. By contrast, there were only 73 and 71 virulence genes detected in O121:H7 strain RM8082 and O121:H10 strain RM10740, respectively (**Figure 3**). This variation in the virulence gene repertoire was mainly due to the difference in the number of genes encoding fimbrial and non-fimbrial adherence factors, toxins, T3SS effectors, as well as genes related to biosynthesis of T3SS and T6SS apparatus (**Supplementary Table 2**).

As observed in the O121:H19 strains, both O121:H7 and O121:H10 strains carried the genes encoding CFA fimbriae (*cfaABCD*), curli fimbriae (*csgDEFG* and *csgBAC*), *E. coli* common pilus (*ecpRABCDE*), *E. coli* laminin-binding fimbriae (*elfADCG*), and hemorrhagic *E. coli* pilus (*hcpCBA*) (**Table 3**). Loss-of-function mutations were detected in various genes, and the mutations included single base insertions or deletions, large DNA fragment deletions, and insertions of transposons or insertion sequences (IS) (**Table 3** and **Supplementary Table 3**). Unlike the O121:H19 strains, neither O121:H10 nor O121:H7 strain carried genes encoding the adhesin intimin (*eae*) or the effector ToxB (*toxB*). Among the five O121 strains examined, only the O121:H7 strain RM8082 carried homologs of K88 fimbriae genes (*faeCDEFHI*) and P fimbriae genes (*papC* and *papH*) (**Table 3**). The O121:H10 strain RM10740 carried an incomplete type I fimbriae gene cluster since homologs of *fimA*, *fimB*, and *fimE* were not detected (**Table 3**). Both plasmid-borne enterohemolysin genes (*hlyCABD*) and the chromosome-borne hemolysin gene (*hlyE*) were detected in the O121:H7 and O121:H10 strains, as observed for the O121:H19 strains. Interestingly, more toxin genes were detected in the O121:H7 strain RM8082, including cytolethal distending toxin genes *cdtABC* and enterotoxin 1 gene *pic*. Although a homolog of heat-labile enterotoxin gene *eltA* was detected, no homolog of *eltB* was identified in the genome of RM8082 (**Supplementary Table 2**).

**Table 3:** Genetic diversity of the adherence genes in STEC O121 strains.

Virulence factors	Related genes	16-9255	2015C-3107	RM8352	RM8082	RM10740
CFA/I fimbriae	*cfaA*	+*	+*	+*	+	+
	*cfaB*	+	+	+	+	+
	*cfaC*	+	+	+	+	+*
	*cfaD/cfaE*	+	+	+	+	+
Curli fimbriae	csgD	+	+	+	+	+
	*csgE*	+	+	+	+	+
	*csgF*	+	+	+	+	+
	*csgG*	+	+	+	+	+*
	*csgA*	+	+	+	+	+
	*csgB*	+	+	+	+	+
	*csgC*	+	+	+	+	+
*E. coli* common pilus (ECP)	*ecpA*	+	+	+	+	+
	*ecpB*	+	+	+	+	+
	*ecpC*	+	+	+	+*	+
	*ecpD*	+	+	+	+	+
	*ecpE*	+	+	+	+*	+
	*ecpR*	+	+	+	+	+*
*E.coli* laminin-binding fimbriae (ELF)	*elfA*	+	+	+	+	+
	*elfC*	+*	+*	+*	+	+
	*elfD*	+*	+*	+*	+	+
	*elfG*	+	+	+	+	+
EaeH/Intimin like adhesin FdeC	*eaeH*	+	+	+	+	+
Hemorrhagic *E.coli* pilus (HCP)	*hcpA*	+	+	+	+	+
	*hcpB*	+	+	+	+	+
	*hcpC*	+	+	+	+	+
Intimin	*eae*	+	+	+	–	–
K88 fimbriae	*faeC*	–	–	–	+	–
	*faeD*	–	–	–	+	–
	*faeE*	–	–	–	+	–
	*faeF*	–	–	–	+	–
	*faeG*	–	–	–	–	–
	*faeH*	–	–	–	+	–
	*faeI*	–	–	–	+	–
	*faeJ*	–	–	–	–	–
P fimbriae	*papA*	–	–	–	–	–
	*papB*	–	–	–	–	–
	*papC*	–	–	–	+	–
	*papD*	–	–	–	–	–
	*papE*	–	–	–	–	–
	*papF*	–	–	–	–	–
	*papG*	–	–	–	–	–
	*papH*	–	–	–	+	–
	*papI*	–	–	–	–	–
	*papJ*	–	–	–	–	–
	*papK*	–	–	–	–	–
	*papX*	–	–	–	–	–
Porcine attaching-effacing associated protein	*paa*	+	+	+	–	+
S fimbriae	*sfaA*	–	–	–	–	–
	*sfaB*	–	–	–	–	–
	*sfaC*	–	–	–	–	–
	*sfaD*	–	–	–	–	–
	*sfaE*	–	–	–	–	–
	*sfaF*	–	–	–	–	–
	*sfaG*	–	–	–	–	–
	*sfaH*	–	–	–	–	–
	*sfaS*	–	–	–	–	–
	*sfaY*	–	–	–	–	–
ToxB	*toxB*	+	+	+	–	–
Type I fimbriae	*fimA*	+	+	+	+	–
	*fimB*	+	+	+	+	–
	*fimC*	+	+	+	+	+
	*fimD*	+	+	+	+	+
	*fimE*	+	+	+	+	–
	*fimF*	+	+	+	+	+
	*fimG*	+	+	+	+	+*
	*fimH*	+	+	+	+	+
	*fimI*	+	+	+	+	+

+, presence; -, absence; +*, carrying a loss-of-function mutation. Detailed information about the mutation is presented in **Supplementary Table 3**.

A total of 34 genes, related to biosynthesis of the T3SS apparatus, were detected in all three O121:H19 strains. In contrast, none of the T3SS apparatus genes were identified in either O121:H7 strain RM8082 or O121:H10 strain RM10740 (**Supplementary Table 2**). Furthermore, the seven LEE-encoded T3SS effectors genes were all detected in the O121:H19 strains. Among the 56 non-LEE encoded T3SS effector genes, a total of 34 were detected in the O121:H19 strains, while only six genes were present in the genomes of O121:H7 and O121:H10 strains (**Supplementary Table 2**). Loss-of-function mutations appeared to be widespread in genes encoding T3SS effectors. Among the 34 genes detected, loss-of-function mutations including single base deletions, single base insertions, large deletions, and nonsense mutations were detected in 10 genes (**Supplementary Table 3**).

Previous studies have documented that there are three T6SS gene clusters reported in *E. coli* (Journet and Cascales, 2016), and among them, T6SS-1 and T6SS-2 are the most common. Like the strain EDL933, no homologs of T6SS-1 genes were identified in any of the O121:H19 strains examined. Homologs of the nine and 11 out of the 27 genes related to T6SS-1 were detected in the O121:H7 strain RM8082 and the O121:H10 strain RM10740, respectively. Among the 22 genes related to T6SS-2, homologs of 15 genes were identified in the O121:H19 strains and the O121:H10 strain RM10740, while only a homolog of *tssI* was identified in the O121:H7 strain RM8082 (**Supplementary Table 2**). Like strain EDL933, none of the O121 strains examined carry any homologs of the T6SS-3 genes.

### 3.4 Comparative analyses of PAIs and fitness islands

Seven PAIs (LEE, LAA, OI-122, OI-57, LPA, HPI, and SE-PAI) contributing to the pathogenicity of enteric pathogens and three GIs (TRI, AFI, and LHR), conferring *E. coli* cells resistance to tellurite, acid, and heat, respectively, were further investigated. The genetic characteristics and sources of the examined PAIs and GIs have been described previously (Carter et al., 2022) and are listed in the **Supplementary Table 1**. Both OI-57 and AFI were detected in all O121 strains examined, while the LEE, OI-122, and TRI were only detected in the O121:H19 strains. On the contrary, the LAA was only detected in the two non-O121:H19 strains.

#### 3.4.1 LEE

**Figure 4:**
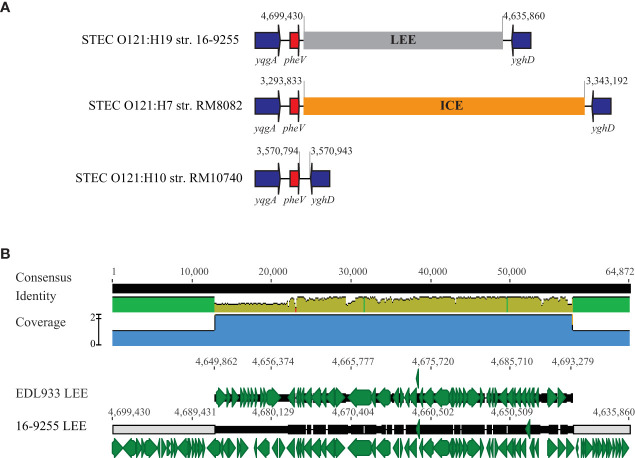
Comparative analysis of LEEs. **(A)** Chromosomal locations of the LEE in STEC O121. The insertion sites of LEE in three O121:H19 strains are identical. This insertion site in the O121:H7 strain RM8082 is occupied by an 83-kb ICE but unoccupied in the O121:H10 strain RM10740. Red arrows indicate the tRNA genes; Blue arrows indicate the bordering genes; Grey block refers to the LEE and the orange block refers to an ICE. Numbers indicate the chromosomal positions. Genes are not drawn to scale. **(B)** Pairwise alignment of EDL933 LEE and the LEE in the O121:H19 strain 16-9255. The identity of consensus is color-coded (Green: 100% identity; Greeny-brown: at least 30% and under 100% identity; Red: below 30% identity). Green arrows represent genes located on the LEEs and numbers indicate the chromosomal positions on the corresponding genome.

The LEE region in O157:H7 strain EDL933 corresponds to the O-island #148 (OI-148) and is inserted downstream of tRNA gene *selC*. A LEE region was identified in all O121:H19 strains, but not in O121:H7 or O121:H10 strains (**Table 4**). The LEEs in the O121:H19 strains were larger in size (~ 64 kb) than the LEE in strain EDL933 (~ 43 kb) and were all located adjacent to tRNA gene *pheV*. This integration site in the O121:H7 strain RM8082 was found occupied by a large Integrative and Conjugative Element (ICE) whereas unoccupied in the O121:H10 strain RM10740 (**Figure 4A**). Sequence comparison of the LEE region in the O121:H19 strains with the EDL933 LEE revealed that O121:H19 contained all key genes related to biosynthesis of T3SS apparatus and the T3SS effectors, as observed for the EDL933 LEE (**Figure 4B**). For O157:H7 EDL933, the LEE-encoded intimin was γ subtype, while all O121:H19 LEEs encode an ϵ subtype intimin. Furthermore, the O121:H19 LEEs carried additional genes encoding transposases, transcriptional regulators, transporters, toxin-antitoxin systems, and hypothetical proteins. The ICE in the O121:H7 strain RM8082 was identified to be ~83 kb and comprised of 98 genes. The known functions encoded by this ICE include heavy metal resistance, transporters, fimbriae, integrating conjugative transfer, DNA modification, protein modification, and transcriptional regulation. BLASTn search of ICE in the NCBI database (nr) revealed homologs of this ICE in strains of *Escherichia* spp. isolated from various sources, including cattle (e.g. strain RHB07-C04), sheep (e.g. strain RHB10-C09), pig (e.g. strain W25K), deer (e.g. strain W49-2), and monkeys (e.g. strain A6). The 15-kb DNA fragment containing the genes related to integrating conjugative function is also present in the ETEC strain UMNK88 (GenBank accession number, CP002729; position: 4,908,729-4,923,578).

**Table 4:** Genetic features and chromosome locations of LEE, LAA, OI-122, and OI-57 in STEC O121 strains.

Strains(Serotypes)	^a^LEE		LAA		^b^OI-122		^c^OI-57	
	Insertion sites	Locations (Size (bp)/%GC)	Insertion sites	Locations (Size (bp)/%GC)	Insertion sites	Locations (Size (bp)/%GC)	Insertion sites	Locations (Size (bp)/%GC)
EDL933 (O157:H7)	*selC*	4,649,862-4,693,279 (43,418/40.9)	NA	ND	*pheV*	3,919,348-3,942,802 (23,455/46.3)	*yciD* (*ompW*)	1,849,324-1,929,825 (80,502/51.4)
16-9255 (O121:H19)	*pheV*	4,699,430-4,635,860 (63,571/43.9)	NA	ND	*pheU*	3,216,904-3,247,701 (30,798/45.1)	*ompW*	1,391,768-1,360,650 (31,119/45.8)
2015C-3107 (O121:H19)	*pheV*	2,209,401-2,274,284 (64,884/44.1)	NA	ND	*pheU*	3,621,869-3,591,073 (30,797/45.1)	*ompW*	49,057-80,373 (31,317/45.9)
RM8352 (O121:H19)	*pheV*	3,255,783-3,192,214 (63,570/43.9)	NA	ND	*pheU*	5,093,450-5,062,654 (30,797/45.1)	*ompW*	1,524,922-1,554,969 (30,048/45.8)
RM8082 (O121:H7)	NA	ND	*selC*	4,121,209-4,225,560 (104,447/46.4)	NA	ND	*ompW*	1,416,252-1,464,227 (47,976/50.8)
RM10740 (O121:H10)	NA	ND	*ileX*	3,174,608-3,056,003 (118,682/49.6)	NA	ND	*ompW*	1,446,065-1,494,042 (47,978/50.5)

a The LEE island in strain EDL933 corresponds to O-island #148 as reported previously (Perna et al., 2001). The LEE regions in the STEC O121:H19 strains were defined by BLASTn search of a database containing all STEC genomes examined in this study using the EDL933 LEE as a query.

b The OI-122 regions in the STEC O121:H19 strains were defined by BLASTn search of a database containing all STEC genomes examined in this study using the OI-122 of the strain EDL933 as a query.

c The OI-57 regions in O121 strains were defined by BLASTn search of a database containing all STEC genomes examined in this study using the EDL933 OI-57 as a query. NA: not applicable; ND: not detected.

#### 3.4.2 LAA

LAA is a large PAI initially identified in STEC O91:H21 strain B2F1 and is present mainly in LEE-negative STEC strains (Montero et al., 2017). The LAA in strain B2F1 is ~81 kb and carries 89 genes, and the known virulence genes are distributed in the four modules, including *sisA* and *hes* on module I, *iha* and *lesP* on module II, *pagC*, *tpsA* and *tpsB* on module III, and *ag43* on module IV (**Supplementary Figure 3A**). The present study identified an incomplete LAA in both O121:H7 and O121:H10 strains but not in any of the O121:H19 strains (**Table 4**). The LAA in strain RM8082 was about 104 kb with an average GC of 46.4%, located adjacent to the tRNA gene *selC*. Among the 91 genes on the RM8082 LAA, 23 exhibited high sequence similarity with the B2F1 LAA genes, including the virulence genes *sisA* and *hes* that are related to attenuation of host immune response and adherence to epithelial cells, respectively (**Supplementary Figure 3B**). Homologs of the module I, III, and IV genes were identified on the RM8082 LAA, but not for any of the virulence genes encoded by the B2F1 LAA module III or IV. Homologs of 13 B2F1 LAA genes were identified in the RM8082 genome but were located outside of the LAA, such as *lesP*, encoding a serine protease autotransporter, *hha*, encoding a hemolysin expression-modulating protein, *btuB*, encoding a TonB-dependent vitamin B12 receptor, and *atoSC* and *atoDAEB* that are involved in short-chain fatty acid degradation. The LAA in strain RM10740 is about 118 kb, located adjacent to the tRNA gene *ileX* (**Table 4**). Among the 139 LAA genes in RM10740, 18 genes exhibit high sequence similarity with the LAA genes in strain B2F1, including the virulence genes *ag43* (locus tag, F1748_15855) (**Supplementary Figure 3C**). Other RM10740 LAA genes with annotated functions included those encoding an antirestriction protein (locus tag: F1748_15870), the DNA repair protein RadC (locus tag: F1748_15875), and a type IV toxin-antitoxin system (locus tags: F1748_15885 and F1748_15890). No other LAA encoded virulence genes were detected in the RM10740 genome.

#### 3.4.3 OI-122

**Figure 5:**
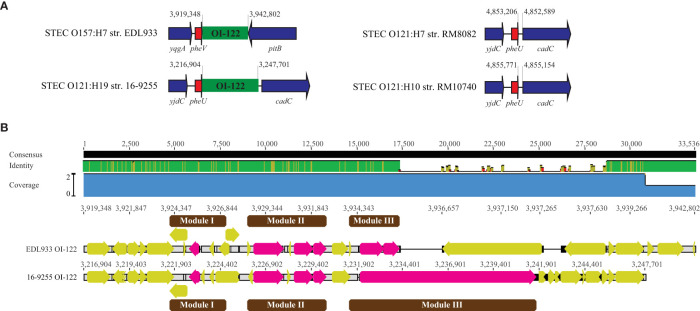
Comparative analysis of OI-122. **(A)** Chromosomal locations of the OI-122 in STEC O121 strains in comparison with the OI-122 in strain EDL933. The insertion sites of OI-122 in the three O121:H19 strains are identical. This insertion site in both O121:H7 strain RM8082 and O121:H10 strain RM10740 is unoccupied. Red arrows represent tRNA genes; Blue arrows represent the bordering genes; Green blocks refer to the OI-122. Numbers indicate the chromosomal positions. Genes are not drawn to scale. **(B)** Pairwise alignment of EDL933 OI-122 with the OI-122 in O121:H19 strain 16-9255. The identity of consensus is color-coded: Green: 100% identity; Greeny-brown: at least 30% and under 100% identity; Red: below 30% identity. Yellow arrows represent genes located on the OI-122s whereas pink arrows represent the OI-122 virulence genes on various modules (Represented by brown blocks). Numbers indicate the chromosomal positions of OI-122 on the corresponding genome.

The OI-122 in EDL933 is 23,455-bp, inserted downstream of the tRNA gene *pheV*. A complete OI-122 was identified in all O121:H19 strains, however, unlike in strain EDL933, it was located downstream of the tRNA *pheU* gene. No homolog of OI-122 was detected in either RM8082 or RM10740. In fact, the integration sites for OI-122 in O121:H19 strains were unoccupied in both non O121:H19 strains (**Table 4** and **Figure 5A**). The EDL933 OI-122 contains three modules, each bordered by MGIs (Konczy et al., 2008). The Module I carries the virulence gene *pagC*, encoding a membrane protein involved in serum resistance; the module II carries genes encoding T3SS effectors EspL, NleB, and NleE; and the module III encodes an Efa1/LifA-like adherence protein. In the present study, the OI-122s in three O121:H19 strains were found to be nearly identical and to exhibit over 85% sequence identity with the OI-122 in strain EDL933 (**Figure 5B**). Both module I and module II were highly conserved in the O121:H19 strains, however, the module III (~10 kb) in the O121:H19 strains was much larger than the module III (~2.8 kb) in strain EDL933 and encoded a full-length Efa1 protein (3,223 aa), which is also known as lymphostatin, a lymphocyte inhibitory virulence factor (Cassady-Cain et al., 2016). In strain EDL933, the coding sequence of *efa1* is truncated by an IS629 at the position 2,133.

#### 3.4.4 PAI OI-57

The OI-57 in strain EDL933 belongs to the genome of prophage CP-933O, carrying virulence genes *adfO* (*paa*) and *ckf*. Additionally, there are two putative virulence genes, one (Locus tag: Z2112) encoding a Clp-like protease and the other one (Locus tag: Z2046) encoding a regulator of cell division (**Supplementary Figure 4A**). Homolog of OI-57, ranging in size from 30,048 to 31,317 bp were identified in all O121:H19 strains (**Table 4**). Like the EDL933 OI-57, the O121:H19 OI-57s were inserted in gene *ompW*, overlapping with the genome of prophage 6, prophage 1, and prophage 6 in strains 16-9255, 2015C-3107, and RM8352, respectively. Among the four virulence genes located on the EDL933 OI-57, all but a homolog of the Clp-like protease was detected on the O121:H19 OI-57 islands (**Supplementary Figure 4B**). Other virulence factors encoded by O121:H19 OI-57s include T3SS effector proteins NleA, NleF, and EspM1.

A 48-kb OI-57 like island was identified in the O121:H7 strain RM8082, overlapping with the genome of prophage 5 (**Table 4**). Among the four EDL933 OI-57 virulence genes, homologs of *ckf* and the gene encoding a regulator of cell division were identified in the RM8082 OI-57 island (**Supplementary Figure 4C**). No homologs of the genes encoding Paa, NleA, NleF, or EspM1 were detected. Similarly, a 48-kb OI-57-like island was detected in the O121:H10 strain RM10740, overlapping with the Stx2e-prophage (prophage 5). Only a homolog of the *ckf* was identified in the RM10740 OI-57 (**Supplementary Figure 4D**). A homolog of the *paa* was detected in the RM10740 genome but located on the large virulence plasmid pEHEC (locus tag: F1748_26265).

#### 3.4.5 TRI

**Figure 6:**
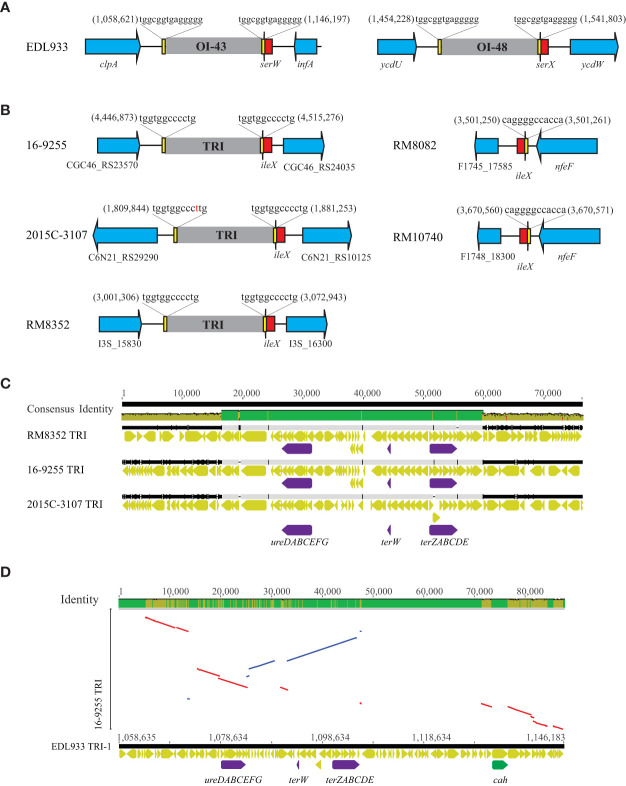
Comparative analyses of TRIs **(A)** Chromosomal locations of the TRIs in O157:H7 strain EDL933. The chromosomal locations of TRIs (OI-43 and OI-48) are indicated by the numbers referencing the start and the end of the bordering Direct Repeats (DRs). Red arrows represent tRNA genes; Yellow blocks refer to the DRs; Blue arrows represent the boarding genes; Grey blocks refer to the TRIs. Genes are not drawn to scale. **(B)** Chromosomal locations of the TRIs in the O121:H19 strains and the corresponding sites in the O121:H7 and O121:H10 strains. The chromosomal locations of TRIs are indicated by the numbers referencing the start and the end of the bordering DRs. This insertion site remains unoccupied in both O121:H7 strain RM8082 and O121:H10 strain RM10740. Red arrows represent tRNA genes; Yellow blocks refer to the DRs; Blue arrows represent the bordering genes; Grey blocks refer to the TRIs. Genes are not drawn to scale and labelled with either the names or the locus tags. **(C)** Multiple alignments of TRIs in the O121:H19 strains. The identity of consensus is color-coded: Green: 100% identity; Greeny-brown: at least 30% and under 100% identity; Red: below 30% identity. Yellow arrows represent the genes on the TRIs whereas purple arrows represent the urease gene cluster and the tellurite resistance genes. **(D)** LASTZ alignment of TRI-1 (OI-43) with the TRI in O121:H19 strain 16-9255. Conserved genes are indicated by lines. Red lines indicate inversions whereas blue lines indicate that the conserved genes are in same orientation in the two genomes. Purple arrows represent the urease genes and the tellurite resistance genes whereas the green arrow refers to *cah*, encoding an autoaggregative adhesin.

The two TRIs in strain EDL933 are located adjacent to tRNA gene *serW* (OI-43), and *serX* (OI-48) (**Table 5**). Both islands are bordered by a 14-bp direct repeat (DR) sequence (Bielaszewska et al., 2011b) (**Figure 6A**). The present study identified a TRI in all three O121:H19 strains, inserted downstream of the tRNA gene *ileX* (**Table 5** and **Figure 6B**). No homologs of TRI were detected in the O121:H7 or the O121:H10 strains. This integration site in both strains was found unoccupied.

**Table 5:** Genetic features and chromosomal locations of the TRI and AFI in STEC O121 strains.

Strains (Serotypes)	^a^Tellurite Resistance Island (TRI)		^b^Acid Fitness Island (AFI)	
	Insertionsites	Chromosomal locations (Size (bp)/%GC)	Bordering Genes	Chromosomal locations (Size (bp)/%GC)
EDL933 (O157:H7)	*serW* (OI-43)	1,058,635-1,146,183 (87,549/48.0)	Unknown gene (locus tag: Z4907) -| |- *yhiA* (locus tag: Z4931)	4,454,268-4,476,943 (22,676/47.6)
	*serX* (OI-48)	1,454,242-1,541,789 (87,548/48.0)		
16-9255 (O121:H19)	*ileX*	4,515,264-4,443,878 (71,387/48.9)	Unknown gene (locus tag: CGC46_RS21305) -| |- *yhiA* (locus tag: CGC46_RS21225)	3,653,961-3,667,580 (13,620/46.0)
2015C-3107 (O121:H19)	*ileX*	1,881,241-1,809,856 (71,386/48.9)	Unknown gene (locus_tag: C6N21_RS15070) -| |- *yhiA* (locus tag: C6N21_RS15150)	2,815,890-2,829,509 (13,620/45.9)
RM8352 (O121:H19)	*ileX*	3,001,318-3,072,931 (71,614/48.9)	Unknown gene (locus tag: I3S_13570) -| |- *yhiA* (locus tag: I3S_13490)	2,580,290-2,566,671 (13,620/45.9)
RM8082 (O121:H7)	NA	ND	Unknown gene (locus tag: F1745_19720) -| |- *yhiA* (F1745_19790)	3,925,122-3,938,741 (13,620/46.0)
RM10740 (O121:H10)	NA	ND	Unknown gene (F1748_20580) -| |- *yhiA* (F1748_20650)	4,125,012-4,138,631 (13,620/46.0)

a The TRIs islands in strain EDL933 correspond to O-island #43 and #48 as reported previously (Perna et al., 2001). The TRI regions in the STEC O121:H19 strains were defined by BLASTn search of a database containing all STEC genomes examined in this study using the OI #48 as a query.

b The AFI regions in the STEC O121:H19 strains were defined by BLASTn search of a database containing all STEC genomes examined in this study using the AFI of the K-12 strain MG1655 as a query as reported previously (Mates et al., 2007). NA: not applicable; ND: not detected.

Like the TRIs in strain EDL933, the TRIs in O121:H19 strains were also bordered by two DRs (**Figure 6B**). The TRIs in the O121:H19 strains displayed over 70% sequence similarity across the entire 70-kb DNA fragment, with greater variations at the beginning of the ~18-kb DNA fragment and the end of the ~13-kb DNA fragment (**Figure 6C**). The central region (~ 40 kb) was observed to be highly conserved and contained the tellurite resistance genes (*terW* and *terZABCDE*) and the urease genes (*ureDABCEFG*). Noteworthy, the *terF* gene was missing in all O121:H19 strains examined and was replaced with a hypothetical gene and an IS3 transposase gene. The O121:H19 TRIs exhibited limited sequence similarity with the EDL933 TRIs (**Figure 6D**). Among the genes with annotated functions, the most conserved ones included the urease genes and tellurite resistance genes. A key virulence gene, *cah*, encoding an auto-aggregative adhesin and located on the EDL933 TRI (Carter et al., 2018), was not present in any of the O121:H19 TRIs.

#### 3.4.6 Other PAIs and GIs

The 37-kb LPA often inserts downstream of tRNA gene *selC*, therefore, it is likely present in LEE-negative strains. This site was found empty in the O121:H19 strains and in the O121:H10 strain RM10740 and occupied by the LAA and LEE in the O121:H7 strain RM8082 and O157:H7 strain EDL933, respectively. Homologs of the LAA virulence genes *espP* and *btuB*, encoding an autotransporter outer membrane protein and the vitamin B12 outer membrane transporter BtuB, respectively, were detected in all O121 genomes but located elsewhere in each corresponding genome. No homologs of HPI, SE-PAI, or LHR were identified in any of the strains examined. The AFIs in all STEC O121 strains were observed to be highly similar to the AFI in *E. coli* K-12 strain MG1655 (**Table 5**), which are genes required for acid resistance (*yhiF*, *yhiD*, *hdeBAD*, *gadE*, *gadW*, *gadX*, and *gadA*) and for an efflux pump (*mdtEF*). Similar to both MG1655 and EDL933, the AFI in all O121 strains was inserted between a hypothetical gene and the gene encoding a cytochrome C peroxidase (*yhiA*). However, unlike the AFI in strain EDL933, the iron acquisition genes *chuSA* and *chuTWXYU* were not detected in any of the O121 genomes examined in this study.

### 3.5 Comparative analysis of the large virulence plasmid pEHEC

The main virulence factors encoded by the plasmid pEHEC in strain EDL933 are enterohemolysin (*hlyCABD*), the type II secretion system (T2SS) (*etpCDEFGHIJKLMNO*), Toxin B (*toxB*), an EHEC-catalase (*katP*), and a serine protease autotransporter (*espP*). While the enterohemolysin genes were conserved in all O121 pEHEC plasmids, the *espP* and *toxB* genes were only detected in pEHEC of the O121:H19 strains and the *katP* was only detected in pEHEC of the O121:H7 strain. No homologs of any T2SS genes were detected in any of O121 pEHECs examined. The RM8082 pEHEC carried a large gene cluster (plasmid position, 10,599-42,528) encoding a type IV conjugative transfer system and a F-type conjugative transfer system. This gene cluster was also present in the RM10740 pEHEC. Some unique genes located on the RM8082 pEHEC included several autotransporter genes, two colicin genes, a hydrolase gene, a sulfite reductase gene, a methyltransferase gene, two transcriptional regulator genes, and the toxin-antitoxin VapBC system. The unique genes carried by the RM10740 pEHEC included the accessory colonization factor AcfC gene, an EamA family transporter gene, a macrolide transporter gene, the gene cluster (*merR* and *merTPCADE*) conferring resistance to mercury (plasmid position: 63,061- 67,023), and the tetracycline resistance genes *tetA* and *tetR*.

### 3.6 Cytotoxicity in STEC O121 strains

**Figure 7:**
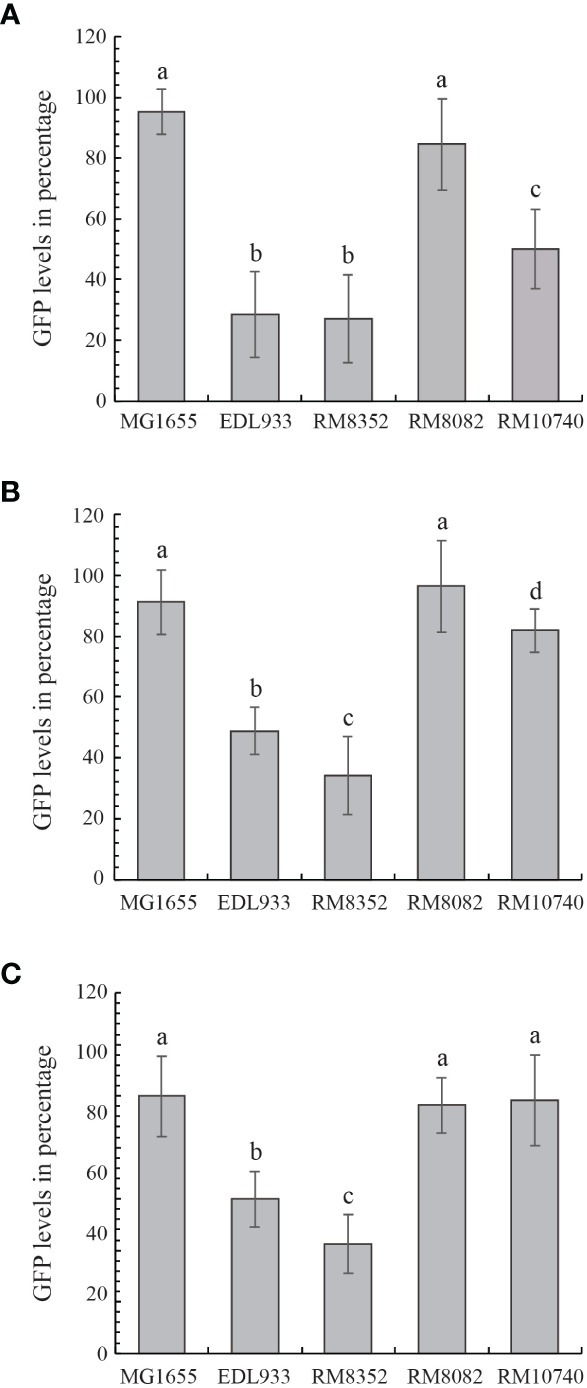
Cytotoxicity of STEC O121 strains. The Stx-mediated cytotoxicity in cultured Vero cell line, Vero-d2EGFP of STEC O121 strains was examined in comparison with K-12 strain MG1655 (negative control) and O157:H7 strain EDL933 (positive control) under high **(A)**, intermediate **(B)** and low **(C)** Stx concentration. High GFP levels represent low Stx cytotoxicity. The cytotoxicity levels in the Vero cells when testing cell-free culture supernatants from the STEC strains were compared using the two-tailed test function in R version 4.2 (R Core Team 2022). STEC strains with significant differences in the Vero cell cytotoxicity level (*P*-value < 0.05) were assigned the different lower-case letters.

The Stx-mediated cytotoxicity of strains RM8352 (O121:H19), RM8082 (O121:H7), and RM10740 (O121:H10) was assessed in the mammalian host Vero cells by using bacterial cell-free culture supernatants and compared with the supernatants from the Stx-negative strain MG1655 and the Stx-positive EHEC prototype strain EDL933. Under the high concentration of the expressed Stx, resulting in low levels of GFP fluorescence in the host Vero cells (**Figure 7A**), the O121:H19 strain RM8352 exhibited comparable cytotoxicity with strain EDL933, which was significantly higher in Stx activity than any of the other strains examined. The O121:H10 strain RM10740 exhibited lower cytotoxicity when compared to EDL933 but still higher toxin activity than that of the control strain MG1655. No significant difference was observed between the O121:H7 strain RM8082 and the control strain MG1655, indicating that Stx expression in strain RM8082 results in minimal cytotoxicity in the mammalian Vero cells. A similar result was observed when less amount of Stxs were used for the cytotoxicity assays, except that O121:H19 strain RM8352 exhibited higher cytotoxicity than that of the strain EDL933 when the Vero cells were intoxicated with the 100-fold dilution of the Stx-containing supernatants (**Figure 7B**). No significant differences in cytotoxicity were detected among the *stx*-negative strain MG1655, the O121:H7 strain RM8082, and the O121:H10 strain RM10740 when testing the 1000-fold dilution of the Stx-containing supernatants (**Figure 7C**).

## 4 Discussion

Our study reported the first complete genomes for STEC strains of serotypes O121:H10 and O121:H7 and demonstrated the distinct evolutionary lineages between them and from the strains of serotype O121:H19. All O121:H19 strains examined in our study carry a genotype of ST655 (Achtman scheme), the main ST reported in the O121:H19 population (Carter et al., 2019; Nishida et al., 2021). The O121:H10 strain RM10740 belongs to ST641, which shares only one common allele, *recA*, with ST655. ST641 appeared to be the main genotype in the serotype O121:H10 population. A search of GenBank revealed 11 draft genomes of *E. coli* O121:H10 strains, which all belong to ST641 (**Supplementary Table 4**). A search of the *E. coli* database at EnteroBase revealed that over 900 strains carry Single Locus Variants (SLVs) of ST641 thus are closely related based on this phylogenetic context to the ST641 strains as suggested previously (Zhou et al., 2020). The main SLVs of ST641 include ST86, ST877, ST1490, ST3858, ST3857, ST1720, and ST3321. Like the ST641 strains including RM10740, most of these strains were isolated from pig feces or processed pig products and were consistent with a previous report that serotype O121:H10 is primarily associated with swine (Kaufmann et al., 2006). The O121:H7 strain RM8082 belongs to ST5082, which does not share any of the seven loci with ST655 and shares only one common allele, *purA*, with ST641 (**Supplementary Table 4**). A total of five draft genomes of O121:H7 strains are available in GenBank as of August 2022, of which, two are clinical isolates, two are bovine isolates, and one was isolated from the lung of a ferret (**Supplementary Table 4**). Like RM8082, three of them belong to ST5082, one belongs to a new ST that differs from ST5082 only at allele *recA*, and one belongs to ST2772. A search of *E. coli* database at EnteroBase revealed 190 strains having SLVs of ST5082, including ST1081, ST1610, ST4380, ST5493, ST1777, ST6345, and ST9463. Like the O121:H7 strain RM8082, most of these strains were isolated from non-clinical sources, including food, wild animals, and livestock.

Our study further revealed distinct virulence potential among the strains belonging to serotypes O121:H19, O121:H10, and O121:H7. The O121:H19 strains represent a hypervirulence lineage as they carry key virulence determinants of EHEC. In fact, this lineage has been suggested to be a third EHEC clone that acquired the EHEC virulence genes independently (Tarr et al., 2002). Like strain EDL933, the prototype of EHEC, all STEC O121:H19 strains examined in this study carry large PAIs such as LEE, OI-122, and OI-57, as well as TRI that confers tellurite resistance and adherence to host epithelial cells. In contrast, none of above PAIs or TRI were present in either O121:H10 or O121:H7 strains, implying a reduced virulence potential in both strains. This distinct virulence genes repertoire is further supported by overall a much smaller number of *E. coli* virulence genes in O121:H7 strain RM8082 and O121:H10 strain RM10740 than any of the O121:H19 strains examined. Additionally, both strains RM8082 and RM10740 carry a Stx subtype that has lower toxicity compared to Stx2a, which is predominantly present in the O121:H19 population (Nishida et al., 2021). As expected, both RM8082 and RM10740 displayed significantly lower Stx-mediated cytotoxicity to Vero cells than the O121:H19 strain RM8352. In fact, the O121:H7 strain RM8082 exhibited minimal cytotoxicity to Vero cells, similar to the control *E. coli* strain that do not harbor any *stx* genes. Future studies are needed to evaluate the pathogenicity potential of RM8082 using other model systems since strains of STEC O121:H7 were reported being isolated from patients and linked to the outbreaks (Trees et al., 2014). In fact, strain RM8082 carries more toxin genes than any of the O121:H19 strains examined, including Cytolethal Distending Toxin (CDT) genes (*cdtABC*) and the serine protease autotransporter (*pic*), which both are known important virulence factors in enteric pathogens (Smith and Bayles, 2006; Abreu et al., 2016; Meza-Segura et al., 2017). Contribution of heat-labile enterotoxin (LT) to the pathogenesis of strain RM8082 remains further investigation as only the gene encoding the subunit A (*eltA*) but not the gene for subunit B (*eltB*) was detected in the genome of RM8082. (**Supplementary Table 2**).

The O121:H10 strain RM10740 exhibits a virulence genes repertoire more similar to that of the O121:H7 strain than the O121:H19 strains. Like RM8082, strain RM10740 does not harbor LEE, OI-122, OI-57, or TRI, but carries a distantly related LAA. However, unlike RM8082, strain RM10740 carries two T6SS gene clusters. The first one is about 28 kb (chromosomal location: 243,168 -271,393) and highly similar to the *E. coli* T6SS-2 (Journet and Cascales, 2016), which is also present in strain EDL933 and the O121:H19 strains. The second one is about 30 kb (chromosomal location: 3,354,985 - 3,385,027) and is highly similar to the *E. coli* T6SS-1 (Journet and Cascales, 2016). This gene cluster was not detected in EDL933 or any O121:H19 strains examined. T6SS plays an important role in the pathogenicity of many enteric pathogens including *E. coli*, especially among strains of EAEC and APEC (Navarro-Garcia et al., 2019). Besides its role in bacterial pathogenesis, T6SS contributes to bacterial survival in diverse environmental niches since it can deliver toxins to both prokaryotic and eukaryotic cells (Ho et al., 2014; Journet and Cascales, 2016).

Stxs, the major virulence factor in STEC, are AB5 toxins that inhibit protein synthesis in eukaryotic cells (Nataro and Kaper, 1998; Melton-Celsa, 2014). Among all the Stx subtypes, Stx2a has been linked to more severe disease and a higher risk of HUS (Fuller et al., 2011; Melton-Celsa, 2014; Kruger and Lucchesi, 2015). It appears that the serotype O121:H19 is associated with the *stx*_2a_ gene. A recent study that investigated the virulence evolution in the STEC O121:H19 population, and among the examined 639 strains, over 95% of the strains carried *stx*_2a_ only, whereas the rest of the population (5%) carried either *stx*_1a_ or both *stx*_1a_ and *stx*_2a_ (Nishida et al., 2021). In contrast, strains of serotype O121:H10 likely are associated with the *stx_2e_* gene. Among the STEC O121:H10 strains reported to date including the five STEC O121:H10 draft genomes deposited in GenBank (**Supplementary Table 4**), all but one carry a *stx*_2e_ gene (Fratamico et al., 2004; Kijima-Tanaka et al., 2005; Fratamico et al., 2008; Bai et al., 2015; Tseng et al., 2015; Baranzoni et al., 2016; Baranzoni et al., 2017; Lee et al., 2018; Zhang et al., 2021). The exception was an ovine isolate and had a *stx*_2a_ gene based on the PCR-RFLP analysis (Zweifel et al., 2004). Similarly, among the STEC O121:H7 strains reported including the three strains with draft genomes available in GenBank (**Supplementary Table 4**), majority of them carry a *stx*_1d_ gene. Although no Vero-cell cytotoxicity was detected for strain RM8082 in our study, *stx*_1d_-positive O121:H7 strains have been isolated from clinical samples and some of them are associated with outbreaks (Trees et al., 2014), implying a pathogenic potential in humans.

In addition to the vast difference in virulence repertoire among the three O121 serotype strains examined, several genes encoding fitness traits differ greatly such as resistance to antibiotics and heavy metals, ability in DNA transferring and acquisition, and transporting and metabolizing of carbon substrates. This divergence could be explained at least in part by their genomic makeups since a considerable amount of strain-specific and accessory genes were detected. For example, among the three environmental strains isolated from the same agricultural region (RM8352, RM8082, and RM10740), about 13%, 12%, and 8% of total CDSs are unique to the strain belonging to O121:H19, O121:H7, and O121:H10, respectively. As expected, this variation is mainly attributed to the difference in the content of MGEs, including prophages, plasmids, GIs/PAIs, and transposable elements. Both O121:H7 strain RM8082 and O121:H10 strain RM10740 carry more plasmids but a smaller number of prophages than the O121:H19 strains. Based on a BLASTn genome search with the ISfinder database, the O121:H10 strain RM10740 contained the greatest number of IS elements with a total of 170, followed by the O121:H19 strains with 136 IS elements, and the O121:H7 strain RM8082 harbored the lowest number of 104 IS elements. Functions encoded by strain-specific genes include, but are not limited to virulence, carbon metabolism, DNA restriction and modification, DNA transfer, and multidrug/heavy metals resistances. It is reasonable to hypothesize that, if not all, some of these functions are niche specific and reflect recent evolution of a particular strain. Although serotype O121:H7 or O121:H10 are not frequently associated with human disease, horizontal gene transfer of *stx*_2e_ for *stx*_2a_ could potentially transform a O121:H10 strain to be a much more virulent strain. Similarly, gene transfer of *stx*_1d_ to a different bacterial host background, such as *E. coli* O8:H9, could transform a strain to be highly pathogenic (Koutsoumanis et al., 2020). Thus, more studies are needed to understand the factors that promote the transfer of virulence genes as well as bacterial factors that contribute to retaining the newly acquired genes. Such information would shed light on the molecular targets for detection of hypervirulent STEC strains.

## Data availability statement

The datasets presented in this study can be found in online repositories. The names of the repository/repositories and accession number(s) can be found in the article/supplementary material.

## Author contributions

Conceptualization: MC. Methodology: AP, BQ, and MC. Software: AP. Data curation: AP, BQ, and NL. Writing—original draft preparation: NL and MC. Writing—review and editing: BQ and MC. Funding acquisition: BQ and MC. Project administration: MC. All authors have read and agreed to the published version of the manuscript. All authors contributed to the article and approved the submitted version.

## Funding

This work was supported by USDA-ARS CRIS projects 2030-42000-052-00D and 2030-42000-055-00D.

## Acknowledgments

We thank Bertram Lee for providing technical assistance with statistical analyses and for critical reading of the manuscript.

## Conflict of interest

The authors declare that the research was conducted in the absence of any commercial or financial relationships that could be construed as a potential conflict of interest.

## Publisher’s note

All claims expressed in this article are solely those of the authors and do not necessarily represent those of their affiliated organizations, or those of the publisher, the editors and the reviewers. Any product that may be evaluated in this article, or claim that may be made by its manufacturer, is not guaranteed or endorsed by the publisher.

## Supplementary material

Supplementary Table 4 Summary of O121:H7 and O121:H10 draft genomes available in GenBank.
