# Supplementary material for: Comparative genomic and phenotypic analyses of the virulence potential in Shiga toxin-producing Escherichia coli O121:H7 and O121:H10
Source: Front Cell Infect Microbiol. 2022 Nov 24;12:1043726. doi: 10.3389/fcimb.2022.1043726 (PMC9729726; doi:10.3389/fcimb.2022.1043726)
Supplement: Supplementary Table 4 — Summary of O121:H7 and O121:H10 draft genomes available in GenBank. [file DataSheet_1.pdf]

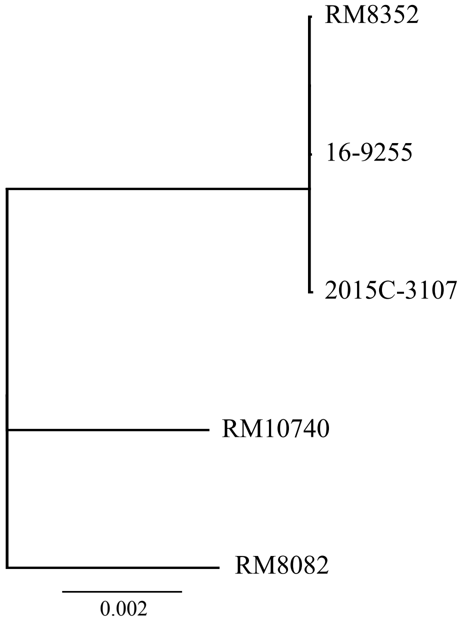

Supplementary Figure 1. The core genes based phylogenetic tree. The core genes of the five genomes were aligned using MUSCLE. The resulting alignments were concatenated to one large alignment that was used as the input for the neighbor-joining algorithm as implemented in the PHYLIP package in Edgar 3.0. The tree shown here was built out of a core of 3762 genes per genome, 18810 in total. The core has 1173829 AA-residues/ bp per genome, 5869145 in total.

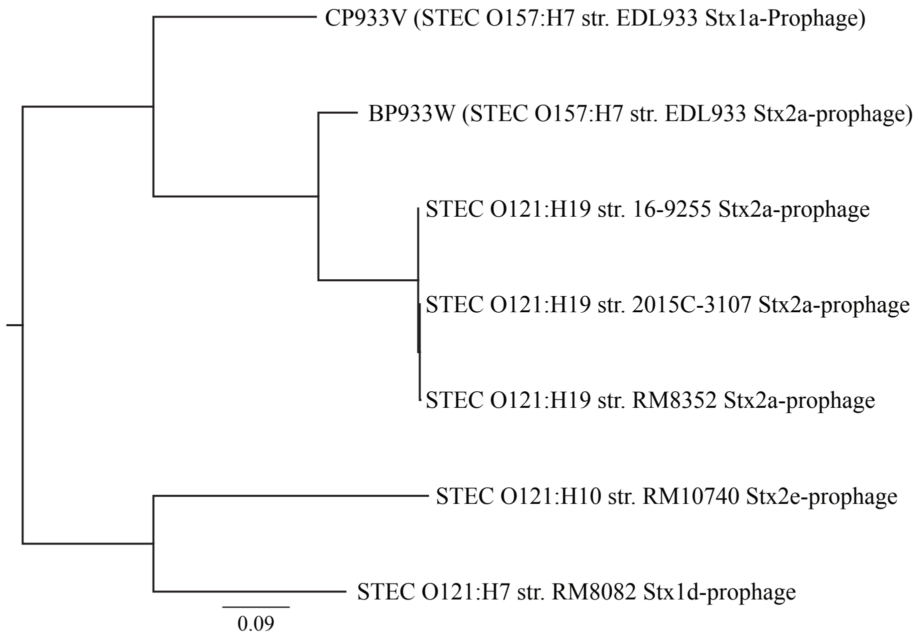

Supplementary Figure 2. Phylogenetic analysis of Stx-prophages in strains of STEC O121. The Stx-prophages were identified using PHASTER. Each Stx-prophage genome was extracted from the corresponding host genome. Sequences of Stx-prophages were aligned using Geneious Alignment with the following parameters: Alignment type: Global alignment with free end gaps; Cost Matrix: 65% similarity; Gap open penalty: 12; Gap extension penalty: 3; and Refinement iterations: 2. A neighbor-joining consensus tree was constructed with the following parameters: Genetic Distance Model, Jukes-Cantor; Resampling Method, bootstrap; and number of replicates, 10,000. The genome sequences of BP-933W, the Stx2a-prophage of STEC O157:H7 strain EDL933, and CP-933V, the Stx1a-prophages of the strain EDL933, were retrieved from GenBank under the accession number AE005174.

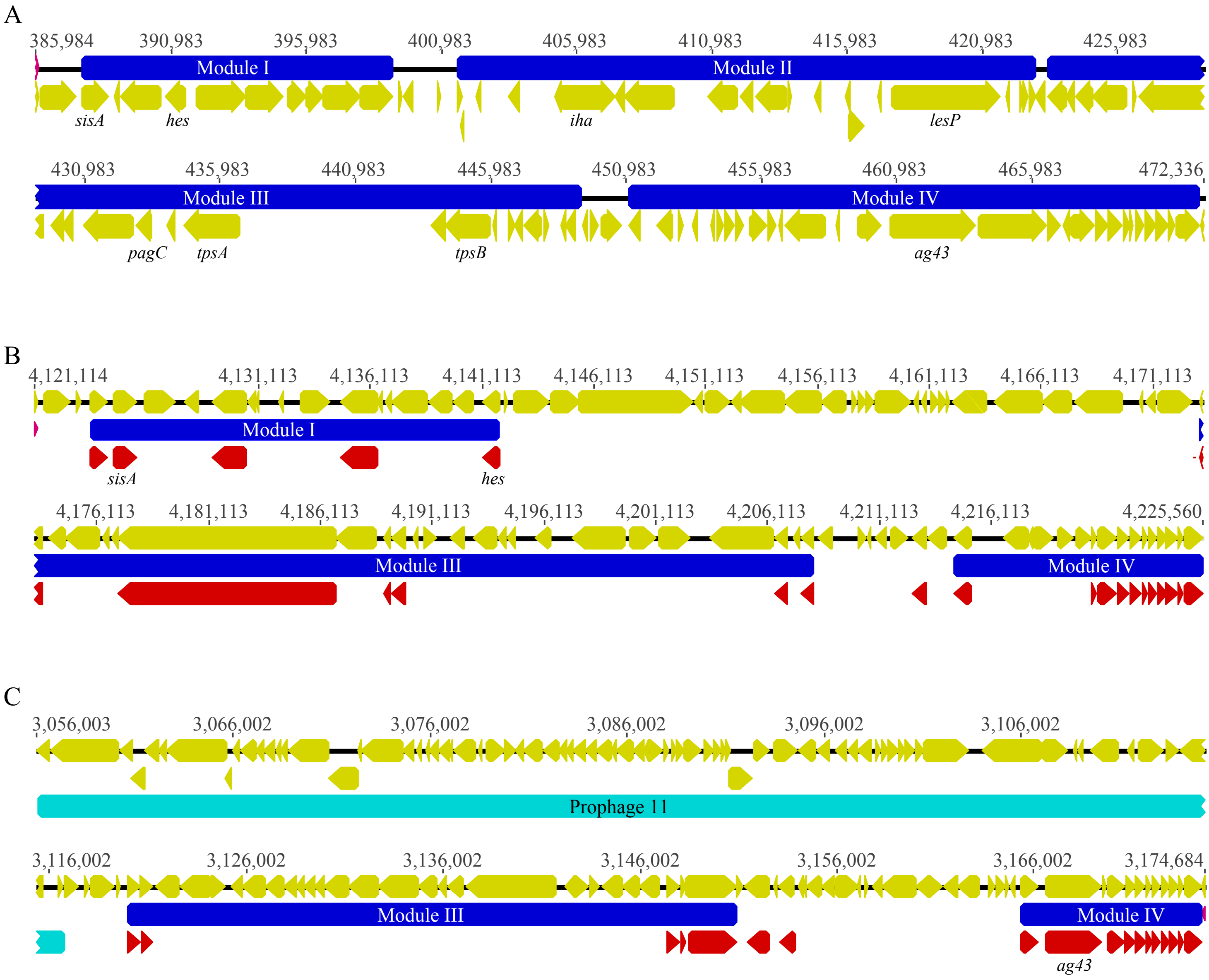

Supplementary Figure 3. Sequence analyses of LAA. Genetic organization of B2F1 LAA (A), LAA in O121:H7 strain RM8082 (B), and LAA in O121:H10 strain RM10740 (C). Blue blocks refer to the modules initially identified in strain B2F1. Numbers indicate the chromosomal location of LAA on the coresponding genome. Yellow arrows represent LAA genes and red arrows represent the conserved B2F1 LAA genes in O121:H7 strain RM8082 (B) and O121:H10 strain RM10740 (C).

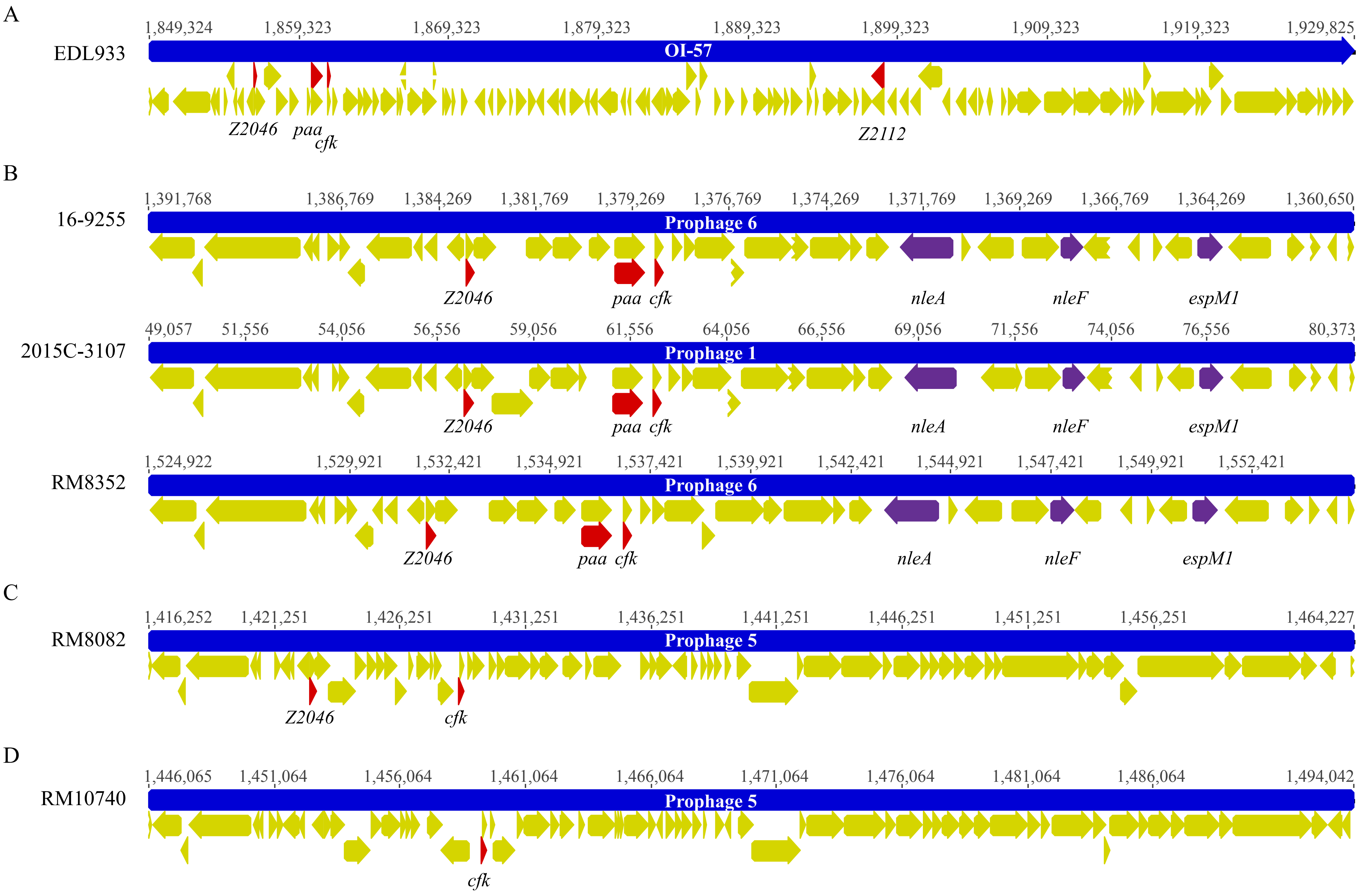

Supplementary Figure 4. Sequence analyses of OI-57. Genetic organization of OI-57 in strain EDL933 (A), in the O121:H19 strains (B), in O121:H7 strain RM8082 (C), and in O121:H10 strain RM10740 (D). Yellow arrows represent OI-57 genes; Red arrows represent the virulence genes initially identified on the EDL933 OI-57. Purple arrows represent additional virulence genes on the O121:H19 OI-57s. Numbers indicate the chromosomal positions on the corresponding genome.

Supplementary Table 1. Characteristics of PAIs and GIs examined in this study

PAIs/GIs	Length (bp)/ %GC	Sources of Query Sequences	GenBank Accession#/Positions	References
Locus of Enterocyte Effacement (LEE)	43,418 / 40.9	STEC O157:H7 str. EDL933	AE005174.2/4,649,862-4,693,279	(Perna et al., 2001)
Locus of Adhesion and Autoaggregation (LAA)	86,353 / 48.6	STEC O91:H21 str. B2F1	AFDQ01000026.1/385,984-472,336	(Montero et al., 2017)
Pathogenicity Island OI-122	23,455 / 46.3	STEC O157:H7 str. EDL933	AE005174.2/3,919,348-3,942,802	(Perna et al., 2001)
Pathogenicity Island OI-57	80,502 / 51.4	STEC O157:H7 str. EDL933	AE005174.2/1,849,324-1,929,825	(Perna et al., 2001)
Locus of Proteolysis Activity (LPA)	37,710 / 47.4	STEC O91:H^- str. 4797/97	AJ278144.1/1-37,710	(Schmidt et al., 2001)
High-Pathogenicity Island (HPI)	36,448 / 56.4	*Yersinia pestis*	AL031866.1/78,113-114,560	(Schubert et al., 1998)
Subtilase Encoding Pathogenicity Island (SE-PAI)	8,058 / 46.2	*E. coli* str. ED32	JQ994271.1/1-8,058	(Michelacci et al., 2013)
Tellurite Resistance Island (TRI)	87,548 / 48.0	STEC O157:H7 str. EDL933	AE005174.2/1,454,242-1,541,789	(Perna et al., 2001)
Acid Fitness Island (AFI)	13,620 / 46.0	*E. coli* str. MG1655	U00096.3/3,653,961-3,667,580	(Mates et al., 2007)
Locus of Heat Resistance (LHR)	14,981 / 62.2	*E. coli* str. P12b	CP002291.1/319,821-304,841	(Mercer et al., 2017)

References:

Mates, A.K., Sayed, A.K., and Foster, J.W. (2007). Products of the *Escherichia coli* acid fitness island attenuate metabolite stress at extremely low pH and mediate a cell density-dependent acid resistance. *Journal of bacteriology* 189(7)**,** 2759-2768.

Mercer, R., Nguyen, O., Ou, Q., McMullen, L., and Gänzle, M.G. (2017). Functional analysis of genes comprising the locus of heat resistance in *Escherichia coli*. *Applied and environmental microbiology* 83(20)**,** e01400-01417.

Michelacci, V., Tozzoli, R., Caprioli, A., Martinez, R., Scheutz, F., Grande, L., et al. (2013). A new pathogenicity island carrying an allelic variant of the Subtilase cytotoxin is common among Shiga toxin producing *Escherichia coli* of human and ovine origin. *Clin Microbiol Infect* 19(3)**,** E149-156. doi: 10.1111/1469-0691.12122.

Montero, D.A., Velasco, J., Del Canto, F., Puente, J.L., Padola, N.L., Rasko, D.A., et al. (2017). Locus of adhesion and autoaggregation (LAA), a pathogenicity island present in emerging Shiga toxin–producing *Escherichia coli* strains. *Scientific reports* 7(1)**,** 1-13.

Perna, N.T., Plunkett, G., 3rd, Burland, V., Mau, B., Glasner, J.D., Rose, D.J., et al. (2001). Genome sequence of enterohaemorrhagic *Escherichia coli* O157:H7. *Nature* 409(6819)**,** 529-533. doi: 10.1038/35054089.

Schmidt, H., Zhang, W.-L., Hemmrich, U., Jelacic, S., Brunder, W., Tarr, P., et al. (2001). Identification and characterization of a novel genomic island integrated at *selC* in locus of enterocyte effacement-negative, Shiga toxin-producing *Escherichia coli*. *Infection and immunity* 69(11)**,** 6863-6873.

Schubert, S., Rakin, A., Karch, H., Carniel, E., and Heesemann, J. (1998). Prevalence of the "high-pathogenicity island" of Yersinia species among *Escherichia coli* strains that are pathogenic to humans. *Infection and immunity* 66(2)**,** 480-485.

Supplementary Table 2. Distribution of *E. coli* virulence genes in STEC O157:H7 strain EDL933 and STEC O121 strains

VFclass	Virulence factors	Genes	Strain/Pathotype	GenBank Accession/Replicon	Locus ID	EDL933	16-9255	2015C-3107	RM8352	*RM8082	RM10740
Adherence	AAF/II fimbriae	*aafA*	*E. coli* O44:H18 str. 042/EAEC	NC_017627/ Plasmid pAA	EC042_pAA048	0	0	0	0	0	0
		aafB			EC042_pAA030	0	0	0	0	0	0
		aafC			EC042_pAA031	0	0	0	0	0	0
		aafD			EC042_pAA046	0	0	0	0	0	0
	AAF/III fimbriae	*aggA*	*E. coli* O104:H4 str. 2011C-3493/StxEAEC	NC_018666/ Plasmid pAA-EA11	O3K_26197	0	0	0	0	0	0
		aggB			O3K_26202	0	0	0	0	0	0
		aggC			O3K_26207	0	0	0	0	0	0
		aggD			O3K_26212	0	0	0	0	0	0
		aggR			O3K_26097	0	0	0	0	0	0
	Afimbrial adhesin AFA-I	*afaA*	*E. coli* str. VR50/UPEC	NZ_CP011134/ Chromosome	ECVR50_3238	0	0	0	0	0	0
		afaB			ECVR50_3237	0	0	0	0	0	0
		afaC			ECVR50_3236	0	0	0	0	0	0
		afaD			ECVR50_3235	0	0	0	0	0	0
		afaE			ECVR50_3233	0	0	0	0	0	0
		draP			ECVR50_3234	0	0	0	0	0	0
	CFA/I fimbriae	*cfaA*	*E. coli* O103:H2 str. 12009/EHEC	NC_013353/ Chromosome	ECO103_3842	0	0	0	0	1	1
		cfaB			ECO103_3841	0	1	1	1	1	1
		cfaC			ECO103_3840	0	1	1	1	1	0
		cfaD/cfaE			ECO103_3839	0	1	1	1	1	1
	Curli fimbriae	*csgD*	*E. coli* O157:H7 str. EDL933/EHEC	NC_002655/ Chromosome	Z1673	1	1	1	1	1	1
		csgE			Z1672	1	1	1	1	1	1
		csgF			Z1671	1	1	1	1	1	1
		csgG			Z1670	1	1	1	1	1	0
		csgA			Z1676	1	1	1	1	1	1
		csgB			Z1675	1	1	1	1	1	1
		csgC			Z1677	1	1	1	1	1	1
	Dispersin	*aap*	*E. coli* O44:H18 str. 042/EAEC	NC_017627/ Plasmid pAA	EC042_pAA055	0	0	0	0	0	0
	E. coli common pilus (ECP)	*ecpA*	*E. coli* O104:H4 str. 2011C-3493/StxEAEC	NC_018658/ Chromosome	O3K_20025	1	1	1	1	1	1
		ecpB			O3K_20030	1	1	1	1	1	1
		ecpC			O3K_20035	1	1	1	1	0	1
		ecpD			O3K_20040	1	1	1	1	1	1
		ecpE			O3K_20045	1	1	1	1	0	1
		ecpR			O3K_20020	1	1	1	1	1	0
	E.coli laminin-binding fimbriae (ELF)	*elfA*	*E.coli* O26:H11 str. 11368/EHEC	NC_013361/ Chromosome	ECO26_1065	1	1	1	1	1	1
		elfC			ECO26_1067	0	0	0	0	1	1
		elfD			ECO26_1066	1	0	0	0	1	1
		elfG			ECO26_1068	0	1	1	1	1	1
	EaeH/Intimin like adhesin FdeC	*eaeH*	*E. coli* O157:H7 str. EDL933/EHEC	NC_002655/ Chromosome	Z0375	1	1	1	1	1	1
	EtpA	*etpA*	*E. coli* O78:H11:K80 str. H10407/ETEC	NC_017724/ Plasmid p948	ETEC_p948_0110	0	0	0	0	0	0
	F1C fimbriae	*focA*		NC_004431/	c1239	0	0	0	0	0	0

		focC	*E. coli* str. CFT073/UPEC	Chromosome	c1241	0	0	0	0	0	0
		focD			c1242	0	0	0	0	0	0
		focF			c1243	0	0	0	0	0	0
		focG			c1244	0	0	0	0	0	0
		focH			c1245	0	0	0	0	0	0
		focI			c1240	0	0	0	0	0	0
	Hemorrhagic *E.coli* pilus (HCP)	*hcpA*	*E. coli* O157:H7 str. EDL933/EHEC	NC_002655/ Chromosome	Z0118	1	1	1	1	1	1
		hcpB			Z0117	1	1	1	1	1	1
		hcpC			Z0116	1	1	1	1	1	1
	Intimin	*eae*	*E. coli* O157:H7 str. EDL933/EHEC	NC_002655/ Chromosome	Z5110	1	1	1	1	0	0
	K88 fimbriae	*faeC*	*E. coli* str. UMNK88/ETEC	NC_017639/ Plasmid pUMNK88_K88	UMNK88_pK887	0	0	0	0	1	0
		faeD			UMNK88_pK888	0	0	0	0	1	0
		faeE			UMNK88_pK889	0	0	0	0	1	0
		faeF			UMNK88_pK8810	0	0	0	0	1	0
		faeG			UMNK88_pK8811	0	0	0	0	0	0
		faeH			UMNK88_pK8812	0	0	0	0	1	0
		faeI			UMNK88_pK8813	0	0	0	0	1	0
		faeJ			UMNK88_pK8814	0	0	0	0	0	0
	P fimbriae	*papA*	*E. coli* str. UM146/AIEC	NC_017632/ Chromosome	UM146_21685	0	0	0	0	0	0
		papB			UM146_21690	0	0	0	0	0	0
		papC			UM146_21675	0	0	0	0	1	0
		papD			UM146_21670	0	0	0	0	0	0
		papE			UM146_21655	0	0	0	0	0	0
		papF			UM146_21650	0	0	0	0	0	0
		papG			UM146_21645	0	0	0	0	0	0
		papH			UM146_21680	0	0	0	0	1	0
		papI			UM146_21695	0	0	0	0	0	0
		papJ			UM146_21665	0	0	0	0	0	0
		papK			UM146_21660	0	0	0	0	0	0
		papX			UM146_21640	0	0	0	0	0	0
	Porcine attaching-effacing associated protein	*paa*	*E. coli* O103:H2 str. 12009/EHEC	NC_013353/ Chromosome	ECO103_1701	1	1	1	1	0	1
	S fimbriae	*sfaA*	*E. coli* str. UM146/AIEC	NC_017632/ Chromosome	UM146_12360	0	0	0	0	0	0
		sfaB			UM146_12365	0	0	0	0	0	0
		sfaC			UM146_12370	0	0	0	0	0	0
		sfaD			UM146_12355	0	0	0	0	0	0
		sfaE			UM146_12350	0	0	0	0	0	0
		sfaF			UM146_12345	0	0	0	0	0	0
		sfaG			UM146_12340	0	0	0	0	0	0
		sfaH			UM146_12330	0	0	0	0	0	0
		sfaS			UM146_12335	0	0	0	0	0	0
		sfaY			UM146_12325	0	0	0	0	0	0
	ToxB	*toxB*	*E. coli* O157:H7 str. EDL933/EHEC	NC_007414/ Plasmid pO157	L7095	1	1	1	1	0	0
	Type I fimbriae	*fimA*	*E. coli* O157:H7	NC_002655/	Z5912	1	1	1	1	1	0

		fimB	str. EDL933/EHEC	Chromosome	Z5910	1	1	1	1	1	0
		fimC			Z5914	1	1	1	1	1	1
		fimD			Z5915	1	1	1	1	1	1
		fimE			Z5911	1	1	1	1	1	0
		fimF			Z5916	1	1	1	1	1	1
		fimG			Z5917	1	1	1	1	1	0
		fimH			Z5918	1	1	1	1	1	1
		fimI			Z5913	1	1	1	1	1	1
Auto-transporter	AIDA-I type	*tibA*	*E. coli* O78:H11:K80 str. H10407/ETEC	NC_017633/ Chromosome	ETEC_2141	0	0	0	0	0	0
	AIDA-I	*aidA*	*E. coli* O25b:H4-ST131/UPEC	NZ_HG941718/ Chromosome	EC958_4121	0	0	0	0	0	0
	AatA	*aatA*	*E. coli* APEC str. O1/APEC	NC_009837/Plasmid pAPEC-O1-ColBM	APECO1_O1CoB M96	0	0	0	0	0	0
	Antigen 43	*agn43*	*E. coli* O104:H4 str. 2011C-3493/StxEAEC	NC_018658/ Chromosome	O3K_04220	0	0	0	0	0	0
	Cah	*cah*	*E. coli* O157:H7 str. EDL933/EHEC	NC_002655/ Chromosome	Z1211	2	0	0	0	0	1
	Contact-dependent inhibition CDI system	*cdiA*	*E. coli* str. UM146/AIEC	NC_017632/ Chromosome	UM146_21955	0	0	0	0	0	0
		cdiB			UM146_21960	0	0	0	0	1	0
	EhaA	*ehaA*	*E. coli* O157:H7 str. EDL933/EHEC	NC_002655/ Chromosome	Z0402	1	1	1	1	0	1
	EhaB	*ehaB*			Z0469	1	1	1	1	1	0
	Enteroaggregative immunoglobulin repeat protein	*air/eaeX*	*E. coli* O44:H18 042/EAEC	NC_017626/ Chromosome	EC042_4012	0	0	0	0	0	0
	EspC	*espC*	*E. coli* O127:H6 str. E2348/69/EPEC	NC_011601/ Chromosome	E2348C_2915	0	0	0	0	0	0
	EspI	*espI*	*E. coli* O103:H2 str. 12009/EHEC	NC_013353/ Chromosome	ECO103_3805	0	1	1	1	0	0
	EspP	*espP*	*E. coli* O157:H7 str. EDL933/EHEC	NC_007414/ Plasmid pO157	L7020	1	1	1	1	0	0
	Pet	*pet*	*E. coli* O104:H4 str. 2011C-3493/StxEAEC	NC_018658/ Chromosome	O3K_23005	0	0	0	0	0	0
	Pic	*pic*			O3K_23140	0	0	0	0	0	0
	Sat	*sat*	*E. coli* O25b:H4-ST131/UPEC	NZ_HG941718/ Chromosome	EC958_3278	0	0	0	0	0	0
	Temperature-sensitive hemagglutinin	*tsh*			EC958_4611	0	0	0	0	1	0
	UpaG adhesin	*upaG/ehaG*			EC958_4009	1	1	1	1	1	1
	UpaH	*upaH*			EC958_1689	0	0	0	0	0	0
	Vacuolating autotransporter gene	*vat*	*E. coli* str. UM146/AIEC	NC_017632/ Chromosome	UM146_15930	0	0	0	0	0	0
Invasion	Invasion of brain endothelial cells (Ibes)	*ibeA*	*E. coli* str. UM146/AIEC	NC_017632/ Chromosome	UM146_22380	0	0	0	0	0	0
		ibeB			UM146_14655	1	1	1	1	0	1
		ibeC			UM146_20030	2	2	2	2	1	1
	Tia/Hek	*tia*			UM146_21625	0	0	0	0	1	0

Iron uptake	Aerobactin siderophore	*iucA*	*E. coli* O104:H4 str. 2011C-3493/StxEAEC	NC_018658/ Chromosome	O3K_23085	0	0	0	0	0	0
		iucB			O3K_23080	0	0	0	0	0	0
		iucC			O3K_23075	0	0	0	0	0	0
		iucD			O3K_23070	0	0	0	0	0	0
		iutA			O3K_23065	0	0	0	0	0	0
	Heme uptake	*chuA*	*E. coli* O157:H7 str. EDL933/EHEC	NC_002655/ Chromosome	Z4911	1	0	0	0	0	0
		chuS			Z4910	1	0	0	0	0	0
		chuT			Z4913	1	0	0	0	0	0
		chuU			Z4918	1	0	0	0	0	0
		chuW			Z4914	1	0	0	0	0	0
		chuX			Z4915	1	0	0	0	0	0
		chuY			Z4917	1	0	0	0	0	0
	Iron-regulated element	*ireA*	*E. coli* CFT073/UPEC	NC_004431/ Chromosome	c5174	0	0	0	0	0	0
	Iron/manganese transport	*sitA*	*E. coli* O44:H18 str. 042/EAEC	NC_017626/ Chromosome	EC042_1521	0	0	0	0	0	0
		sitB			EC042_1520	0	0	0	0	0	0
		sitC			EC042_1519	0	0	0	0	0	0
		sitD			EC042_1518	0	0	0	0	0	0
	Salmochelin siderophore	*iroB*	*E. coli* str.UM146/AIEC	NC_017632/ Chromosome	UM146_12295	1	0	0	0	0	0
		iroC			UM146_12300	0	0	0	0	0	0
		iroD			UM146_12305	0	0	0	0	0	0
		iroE			UM146_12310	0	0	0	0	0	0
		iroN			UM146_12315	0	0	0	0	0	0
	Yersiniabactin siderophore	*fyuA*	*E. coli* O45:K1:H7 str. S88/NMEC	NC_011742/ Chromosome	ECS88_2043	0	0	0	0	0	0
		irp1			ECS88_2038	0	0	0	0	0	0
		irp2			ECS88_2037	0	0	0	0	0	0
		ybtA			ECS88_2036	0	0	0	0	0	0
		ybtE			ECS88_2041	0	0	0	0	0	0
		ybtP			ECS88_2035	0	0	0	0	0	0
		ybtQ			ECS88_2034	0	0	0	0	0	0
		ybtS			ECS88_2032	0	0	0	0	0	0
		ybtT			ECS88_2040	0	0	0	0	0	0
		ybtU			ECS88_2039	0	0	0	0	0	0
		ybtX			ECS88_2033	0	0	0	0	0	0
LEE-encoded T3SS effectors	EspB	*espB*	*E. coli* O157:H7 str. EDL933/EHEC	NC_002655/ Chromosome	Z5105	1	1	1	1	0	0
	EspF	*espF*			Z5100	1	1	1	1	0	0
	EspG	*espG*			Z5142	1	1	1	1	0	0
	EspH	*espH*			Z5115	1	1	1	1	0	0
	Mitochondria-associated protein Map	*map*			Z5113	1	1	1	1	0	0
	SepZ/EspZ	*EspZ*			Z5122	1	1	1	1	0	0
	Tir	*tir*			Z5112	1	1	1	1	0	0
Non-LEE encoded T3SS effectors	Cell-cycle-inhibitory factor Cif	*cif*	*E. coli* O127:H6 str. E2348/69/EPEC	NC_011601/ Chromosome	E2348C_0719	0	0	0	0	0	0
	EspFu/TccP (Tir cytoskeleton coupling protein)	*espFu/tccP*	*E. coli* O157:H7 str. EDL933/EHEC	NC_002655/ Chromosome	Z3072	1	1	1	1	0	0

	EspG2 (EPEC EspC island)	*espG2*	*E. coli* O127:H6 str. E2348/69/EPEC	NC_011601/ Chromosome	E2348C_2916	0	0	0	0	0	0
	EspJ	*espJ*	*E. coli* O157:H7 str. EDL933/EHEC	NC_002655/ Chromosome	Z3071	1	2	2	2	0	0
	EspK	*espK*			Z1829	1	3	3	3	0	0
	EspL1	*espL1*			Z2749	1	0	0	0	0	0
	EspL2	*espL2*			Z4326	1	1	1	1	0	0
	EspL4	*espL4*			Z5608	1	0	0	0	0	0
	EspM1	*espM1*			Z2565	1	1	1	1	0	0
	EspM2	*espM2*			Z3918	1	1	1	1	0	0
	EspN	*espN*			Z1824	0	0	0	0	0	0
	EspO1-1	*espO1-1*	*E. coli* O157:H7 str. Sakai/EHEC	NC_002695	ECs1567	1	1	1	1	0	0
	EspO1-2	*espO1-2*			ECs1821	1	2	2	2	0	0
	EspR1	*espR1*	*E. coli* O157:H7 str. EDL933/EHEC	NC_002655/ Chromosome	Z2242	1	0	0	0	1	1
	EspR3	*espR3*			Z3023	1	0	0	0	0	0
	EspR4	*espR4*			Z3026	1	0	0	0	0	0
	EspV	*espV*			Z1387	1	1	0	1	0	0
	EspW	*espW*			Z3920	1	1	1	1	0	0
	EspX1	*espX1*			Z0025	1	0	0	0	0	0
	EspX2	*espX2*			Z1019	1	0	0	0	0	0
	EspX4	*espX4*			Z5636	1	0	0	0	0	0
	EspX5	*espX5*			Z5665	1	0	0	0	1	1
	EspX6	*espX6*			Z5935	1	0	0	0	0	0
	EspX7	*espX7*			Z1822	1	1	1	1	0	0
	EspY1	*espY1*			Z0065	1	0	0	0	0	0
	EspY2	*espY2*			Z0078	1	0	0	0	0	0
	EspY3	*espY3*			Z0521	1	0	0	0	0	0
	EspY4	*espY4*			Z5211	1	0	0	0	0	0
	EspY5	*espY5*			Z5214	1	0	0	0	0	0
	LifA/Efa1	*lifA/efa1*			Z4332	1	0	0	0	0	0
	LifA/Efa2	*lifA/efa1*			Z4333	1	1	1	1	0	0
	NleA	*nleA*			Z6024	1	1	1	1	0	0
	NleB1	*nleB1*			Z4328	1	1	1	1	0	0
	NleB2-1	*nleB2-1*	*E. coli* O103:H2 str. 12009/EHEC	NC_013353/ Chromosome	ECO103_2227	1	0	0	0	0	0
	NleB2-2	*nleB2-2*			ECO103_0801	0	0	0	0	0	0
	NleC	*nleC*	*E. coli* O157:H7 str. EDL933/EHEC	NC_002655/ Chromosome	Z0986	1	2	3	3	0	0
	NleD	*nleD*			Z0990	1	0	0	0	0	0
	NleE-1	*nleE-1*			Z4329	1	1	1	1	0	0
	NleE-2	*nleE-2*	*E. coli* O127:H6 str. E2348/69/EPEC	NC_011601/ Chromosome	E2348C_1080	0	0	0	0	0	0
	NleF	*nleF*	*E. coli* O157:H7 str. EDL933/EHEC	NC_002655/ Chromosome	Z6020	1	1	1	1	0	0
	NleG-1	*nleG-1*			Z6010	1	2	2	2	0	0
	NleG-2	*nleG-2*	*E. coli* O55:H7 str. CB9615/EPEC	NC_013941/ Chromosome	G2583_0341	0	0	0	0	0	0
	NleG-3	*nleG-3*	*E. coli* O157:H7 str. EC4115/EHEC	NC_011353/ Chromosome	ECH74115_5546	0	2	2	2	0	0
	NleG2-2	*nleG2-2*	*E. coli* O157:H7 str. EDL933/EHEC	NC_002655/ Chromosome	Z2339	1	0	0	0	0	0
	NleG2-3	*nleG2-3*			Z2149	1	1	1	1	0	0

	NleG2-4	*nleG2-4*			Z2075	1	1	1	1	0	0
	NleG5-1	*nleG5-1*			Z2337	1	1	1	1	0	0
	NleG5-2	*nleG5-2*			Z2151	1	0	0	0	0	0
	NleG6-1	*nleG6-1*			Z2338	1	2	2	2	0	0
	NleG6-2	*nleG6-2*			Z2150	1	0	0	0	0	0
	NleG6-3	*nleG6-3*			Z3921	1	0	0	0	0	0
	NleG7	*nleG7*			Z2077	1	0	0	0	0	0
	NleG8-2	*nleG8-2*			Z3919	1	1	1	1	0	0
	NleH1-1	*nleH1-1*			Z0989	1	0	0	0	0	0
	NleH1-2	*nleH1-2*			Z6021	1	1	1	1	0	0
	TccP2	*tccP2*			Z1385	1	0	0	0	0	0
Secretion system	T6SS-3	*aaiA*	*E. coli* O104:H4 str. 2011C-3493/StxEAEC	NC_018658/ Chromosome	O3K_04355	0	0	0	0	0	0
		aaiB			O3K_04360	0	0	0	0	0	0
		aaiC/hcp			O3K_04365	0	0	0	0	0	0
		aaiD			O3K_04370	0	0	0	0	0	0
		aaiE			O3K_04380	0	0	0	0	0	0
		aaiF			O3K_04385	0	0	0	0	0	0
		aaiH			O3K_04395	0	0	0	0	0	0
		aaiI			O3K_04400	0	0	0	0	0	0
		aaiJ			O3K_04405	0	0	0	0	0	0
		aaiK			O3K_04410	0	0	0	0	0	0
		aaiL			O3K_04415	0	0	0	0	0	0
		aaiM			O3K_04420	0	0	0	0	0	0
		aaiN			O3K_04425	0	0	0	0	0	0
		clpV/aaiP			O3K_04435	0	0	0	0	0	0
		icmF/aaiO			O3K_04430	0	0	0	0	0	0
		vgrG			O3K_04390	0	0	0	0	0	0
	ABC transporter for dispersin	*aatA*	*E. coli* O104:H4 str. 2011C-3493/StxEAEC	NC_018666/ Chromosome	O3K_26477	0	0	0	0	0	0
		aatB			O3K_26472	0	0	0	0	0	0
		aatC			O3K_26467	0	0	0	0	0	0
		aatD			O3K_26462	0	0	0	0	0	0
		aatP			O3K_26482	0	0	0	0	0	0
	T6SS-2	*tssF*	*E. coli* O45:K1:H7 str. S88/NMEC	NC_011742/ Chromosome	ECS88_0243	1	1	1	1	0	1
		tssI			ECS88_1552	0	0	0	0	1	0
		aec11			ECS88_0251	0	0	0	0	0	0
		aec14			ECS88_0249	0	0	0	0	0	0
		aec15			ECS88_0248	3	3	3	0	0	3
		aec16			ECS88_0247	1	1	1	1	0	1
		aec17			ECS88_0246	0	0	0	0	0	0
		aec18			ECS88_0245	0	0	0	0	0	0
		aec19			ECS88_0244	0	0	0	0	0	0
		aec22			ECS88_0242	1	1	0	1	0	1
		aec23			ECS88_0241	1	1	1	1	0	1
		aec24			ECS88_0240	1	1	1	1	0	1
		aec25			ECS88_0239	1	0	0	0	0	1
		aec26			ECS88_0238	1	1	1	1	0	1
		aec27/clpV			ECS88_0237	1	1	1	1	0	1

		aec28			ECS88_0236	1	1	1	1	0	1
		aec29			ECS88_0235	0	1	1	1	0	1
		aec30			ECS88_0234	1	1	1	1	0	1
		aec31			ECS88_0233	1	1	1	1	0	1
		aec32			ECS88_0232	1	1	1	1	0	1
		aec7			ECS88_0255	0	0	0	0	0	0
		aec8			ECS88_0254	0	0	0	0	0	0
	T3SS	ND	*E. coli* O157:H7 str. EDL933/EHEC	NC_002655/ Chromosome	Z5102	1	1	1	1	0	0
		ND			Z5121	1	1	1	1	0	0
		cesAB			Z5138	1	1	1	1	0	0
		ND			Z5139	1	1	1	1	0	0
		ND			Z5143	1	1	1	1	0	0
		cesD2			Z5104	1	1	1	1	0	0
		cesD			Z5127	1	1	1	1	0	0
		cesF			Z5114	1	1	1	1	0	0
		cesT			Z5111	1	1	1	1	0	0
		escC			Z5126	1	1	1	1	0	0
		escD			Z5109	1	1	1	1	0	0
		escF			Z5103	1	1	1	1	0	0
		escI			Z5123	1	1	1	1	0	0
		escJ			Z5124	1	1	1	1	0	0
		escK			Z5137	1	1	1	1	0	0
		escL			Z5136	1	1	1	1	0	0
		escN			Z5119	1	1	1	1	0	0
		escO			Z5118	1	1	1	1	0	0
		escP			Z5117	1	1	1	1	0	0
		escR			Z5135	1	1	1	1	0	0
		escS			Z5134	1	1	1	1	0	0
		escT			Z5133	1	1	1	1	0	0
		escU			Z5132	1	1	1	1	0	0
		escV			Z5120	1	1	1	1	0	0
		espA			Z5107	1	1	1	1	0	0
		espB			Z5105	1	1	1	1	0	0
		espD			Z5106	1	1	1	1	0	0
		etgA			Z5131	1	1	1	1	0	0
		glrA			Z5128	1	1	1	1	0	0
		glrR			Z5129	1	0	0	0	0	0
		ler			Z5140	1	1	1	1	0	0
		sepD			Z5125	1	1	1	1	0	0
		sepL			Z5108	1	1	1	1	0	0
		sepQ			Z5116	1	1	1	1	0	0
	T6SS-1	*tssB*	*E. coli* O44:H18 str. 042/EAEC	NC_017626/ Chromosome	EC042_4524	0	0	0	0	1	1
		tssC			EC042_4525	0	0	0	0	1	1
		tssK			EC042_4526	0	0	0	0	1	1
		tssL			EC042_4527	0	0	0	0	1	0
		ND			EC042_4528	0	0	0	0	0	1
		hcp			EC042_4529	0	0	0	0	0	1

		tssH			EC042_4530	0	0	0	0	0	1
		ND			EC042_4531	0	0	0	0	0	0
		ND			EC042_4532	0	0	0	0	0	0
		vgrG			EC042_4533	0	0	0	0	0	3
		ND			EC042_4534	0	0	0	0	0	0
		ND			EC042_4535	0	0	0	0	0	0
		ND			EC042_4536	0	0	0	0	0	0
		ND			EC042_4537	0	0	0	0	0	0
		ND			EC042_4538	0	0	0	0	0	0
		tssM			EC042_4539	0	0	0	0	1	0
		tssA			EC042_4540	0	0	0	0	0	0
		ND			EC042_4541	0	0	0	0	0	0
		tssF			EC042_4542	0	0	0	0	1	1
		tssG			EC042_4543	0	0	0	0	1	1
		tssJ			EC042_4544	0	0	0	0	1	1
		tssE			EC042_4545	0	0	0	0	1	1
		ND			EC042_4546	0	0	0	0	0	0
		ND			EC042_4547	0	0	0	0	0	0
		ND			EC042_4548	0	0	0	0	0	0
		ND			EC042_4549	0	0	0	0	0	0
		ND			EC042_4550	0	0	0	0	0	0
Toxin	Alpha-hemolysin	*hlyA*	*E. coli* O157:H7 str. EDL933/EHEC	NC_007414/ Plasmid pO157	L7048	1	1	1	1	1	1
		hlyB			L7049	1	1	1	1	0	1
		hlyC			L7047	1	1	1	0	1	1
		hlyD			L7050	1	1	1	0	1	1
	Colicin-like Usp	*hcp*	*E. coli* str. UM146/AIEC	NC_017632/ Chromosome	UM146_23345	0	0	0	0	0	0
	Cytolethal distending toxin	*cdtA*	*E. coli* O18:K1:H7 str. IHE3034/NMEC	NC_017628/ Chromosome	ECOK1_2089	0	0	0	0	1	0
		cdtB			ECOK1_2088	0	0	0	0	1	0
		cdtC			ECOK1_2087	0	0	0	0	1	0
	Cytotoxic necrotizing factor 1	*cnf1*	*E. coli* str. UM146/AIEC	NC_017632/ Chromosome	UM146_21825	0	0	0	0	0	0
	Enterotoxin 1	*pic*	*E. coli* O44:H18 str. 042/EAEC	NC_017626/ Chromosome	EC042_4593	0	0	0	0	2	0
	Enterotoxin SenB/TieB	*senB*	*E. coli* str. UM146/AIEC	NC_017630/ Chromosome	UM146_24581	0	0	0	0	0	0
	Heat-labile enterotoxin	*eltA*	*E. coli* O78:H11:K80 str. H10407/ETEC	NC_017722/ Plasmid p666	ETEC_p666_0660	0	0	0	0	1	0
		eltB			ETEC_p666_0650	0	0	0	0	0	0
	Hemolysin/ cytolysin A	*hlyE/clyA*	*E. coli* O157:H7 str. EDL933/EHEC	NC_002655/ Chromosome	Z1944	1	1	1	1	0	1
	Shiga toxin	*stx1A*	*E. coli* O157:H7 str. EDL933/EHEC	NC_002655/ Chromosome	Z3344	1	0	0	0	1	0
		stx1B			Z3343	1	0	0	0	1	0
		stx2A	*E. coli* O157:H7 str. EDL933/EHEC	NC_002655/ Chromosome	Z1464	1	1	1	1	0	1
		stx2B			Z1465	1	1	1	1	0	1

0: absence or carrying a loss-of-function mutation; 1: WT gene; >1: the copy number of the WT gene; NA: not available; ND: Undetermined; *There are additional four T6SS genes identified (F1745_01065, F1745_01100, F1745_01105, and F1745_01045) in the genome of RM8082.

Supplementary Table 3. Loss-of-function mutations in *E. coli* virulence genes

VFclass	Virulence Factors	Genes	EDL933	16-9255	2015C-3107	RM8352	RM8082	RM10740
Adherence	CFA/I fimbriae	*cfaA*	ND	Large deletion	Nonsense mutation	Large deletion	WT	WT
		cfaC	ND	WT	WT	WT	WT	IS insertion
	Curli fimbriae	*csgG*	WT	WT	WT	WT	WT	IS insertion
	E. coli common pilus (ECP)	*ecpC*	WT	WT	WT	WT	Point deletion	WT
		ecpE	WT	WT	WT	WT	Point deletion	WT
		ecpR	WT	WT	WT	WT	WT	Point deletion
	E.coli laminin-binding fimbriae (ELF)	*elfC*	Point deletion	Large deletion	Large deletion	Large deletion	WT	WT
		elfD	WT	Point deletion	Point deletion	Point deletion	WT	WT
		elfG	Nonsense mutation	WT	WT	WT	WT	WT
	Type I fimbriae	*fimG*	WT	WT	WT	WT	WT	Point deletion
Auto-transporter	Contact-dependent inhibition CDI system	*cdiA*	ND	ND	ND	ND	Nonsense mutation	ND
	EhaB	*ehaB*	WT	WT	WT	WT	WT	IS insertion
Invasion	Invasion of brain endothelial cells (Ibes)	*ibeB*	WT	WT	WT	WT	Point deletion	WT
Non-LEE encoded T3SS effectors	EspL1	*espL1*	WT	Large deletion	Large deletion	Large deletion	Nonsense mutation	Nonsense mutation
	EspL4	*espL4*	WT	Point deletion	Point deletion	Point deletion	Large deletion	Large deletion
	EspN	*espN*	Point deletion	Point deletion	Point deletion	Point deletion	ND	ND
	EspR1	*espR1*	WT	Point insertion	Point insertion	Point insertion	WT	WT
	EspV	*espV*	WT	WT	Point deletion	WT	ND	ND
	EspX1	*espX1*	WT	Point deletion	Point deletion	Point deletion	Point deletion	Point deletion
	EspX4	*espX4*	WT	Point deletion	Point deletion	Point deletion	Point deletion	Point deletion
	EspX5	*espX5*	WT	Point insertion	Point insertion	Point insertion	WT	WT
	NleC	*nleC*	WT	Point deletion	WT	WT	ND	ND
	NleG7	*nleG7*	WT	Point deletion	Point deletion	Point deletion	ND	ND
T3SS	GlrR	*glrR*	WT	Point insertion	Point insertion	Point insertion	ND	ND
Secretion system	T6SS-2	*aec15*	WT	WT	WT	Point deletion	ND	WT
		aec18	Large deletion	Large deletion	Large deletion	Large deletion	ND	Large deletion
		aec22	WT	WT	IS insertion	WT	ND	WT
		aec25	WT	Large deletion	Large deletion	Large deletion	ND	WT
	T6SS-1	*hcp*	ND	ND	ND	ND	ND	WT
		tssH	ND	ND	ND	ND	ND	WT
		tagK	ND	ND	ND	ND	Nonsense mutation	ND
Toxin	Alpha-hemolysin	*hlyB*	WT	WT	WT	WT	Point deletion	WT
	Hemolysin/cytolysin A	*hlyE*	WT	WT	WT	WT	Point deletion	WT

ND: Not detected; WT: wild-type gene

Supplementary Table 4. Characteristics of O121:H7 and O121:H10 draft genomes available in GenBank[1]

Strain	ST	MLST alleles[2]							*stx* gene[3]	Serotype	Sources	GenBank Accession
		adk	*fumC*	*gyrB*	*icd*	*mdh*	*purA*	*recA*				
2009C-3299	5082	6	4	3	18	11	8	2	stx_{1d}	O121:H7	Clinical sample/USA	NZ_JHHC00000000.1
CAP25	5082	6	4	3	18	11	8	2	ND	O121:H7	Cattle/Canada	NZ_JAAKCG000000000.1
MOD1-EC5968	2772	6	6	3	26	7	8	6	ND	O121:H7	Mustela putorius fur/USA	NZ_NMGM00000000.1
PNUSAE104648	5082	6	4	3	18	11	8	2	stx_{1d}	O121:H7	Clinical sample/USA	ABGUGF000000000.1
CFSAN026842	New	6	4	3	18	11	8	512	stx_{1d}	O121:H7	Cattle/USA	LDCX00000000.1
CE2-R3-15-1003	641	9	6	33	131	24	8	7	stx_{2e}	O121:H10	Ground pork/Canada	DABZKA000000000.1
CE-R3-15-0020	641	9	6	33	131	24	8	7	stx_{2e}	O121:H10	Ground pork/Canada	DABZJT000000000.1
CFIAFB20150322	641	9	6	33	131	24	8	7	stx_{2e}	O121:H10	Food/Canada	DAAYYV000000000.1
FWSEC0404	641	9	6	33	131	24	8	7	stx_{2e}	O121:H10	River water/Canada	NZ_RROA00000000.1
MOD1-EC5646	641	9	6	33	131	24	8	7	ND	O121:H10	Feces (Sus domesticus)/USA	NZ_NLJM00000000.1
MOD1-EC5648	641	9	6	33	131	24	8	7	ND	O121:H10	Feces (Sus domesticus)/USA	NODS00000000.1
MOD1-EC5649	641	9	6	33	131	24	8	7	ND	O121:H10	Feces (Sus domesticus)/USA	NLJK00000000.1
MOD1-EC5650	641	9	6	33	131	24	8	7	ND	O121:H10	Feces (Sus domesticus)/USA	NLJJ00000000.1
MOD1-EC5659	641	9	6	33	131	24	8	7	ND	O121:H10	Feces (Sus domesticus)/USA	NLJA00000000.1
MOD1-EC6915	641	9	6	33	131	24	8	7	ND	O121:H10	Feces (Sus domesticus)/USA	NMNP00000000.1
PNUSAE79614	641	9	6	33	131	24	8	7	stx_{2e}	O121:H10	Clinical sample/USA	ABBVFG000000000.1

[1] Available as of August 2022. [2]The MLST profile of ST655 is adk_100, fumC_23, gyrB_68, icd_45, mdh_1, purA_35, recA_7. [3]ND: not detected.
